# Five-Coordinated Geometries from Molecular Structures to Solutions in Copper(II) Complexes Generated from Polydentate-*N*-Donor Ligands and Pseudohalides

**DOI:** 10.3390/molecules25153376

**Published:** 2020-07-25

**Authors:** Franz A. Mautner, Roland C. Fischer, Ana Torvisco, Maher M. Henary, Febee R. Louka, Salah S. Massoud, Nahed M. H. Salem

**Affiliations:** 1 Institut für Physikalische und Theoretische Chemie, Technische Universität Graz, Stremayrgasse 9, A-8010 Graz, Austria; 2 Institut für Anorganische Chemie, Technische Universität Graz, Stremayrgasse 9, A-8010 Graz, Austria; roland.fischer@tugraz.at (R.C.F.); ana.torviscogomez@tugraz.at (A.T.); 3 Department of Chemistry and Biochemistry, University of California, Los Angeles, CA 90095-1569, USA; henary@chem.ucla.edu; 4 Department of Chemistry, University of Louisiana at Lafayette, P.O. Box 43700, Lafayette, LA 70504, USA; febee.louka@louisiana.edu; 5 Department of Chemistry, Faculty of Science, Alexandria University, Moharam Bey, Alexandria 21511, Egypt; nahed_s@yahoo.com

**Keywords:** copper, crystal structure, isothiocyanate, azide, dicyanamide, five-coordinated, UV-vis spectra

## Abstract

A novel series of mononuclear five-coordinated pseudohalido-Cu(II) complexes displaying distorted square bipyramidal: [Cu(L^1^)(NCS)_2_] (**1**), [Cu(L^2^)(NCS)_2_] (**2**) and [Cu(L^3^)(NCS)]ClO_4_ (**5**) as well as distorted trigonal bipyramidal: [Cu(isp_3_tren)(N_3_)]ClO_4_ (**3**), [Cu(isp_3_tren)(dca)]ClO_4_ (**4**) and [Cu(tedmpza)(dca)]ClO_4_·0.67H_2_O (**6**) geometries had been synthesized and structurally characterized using X-ray single crystal crystallography, elemental microanalysis, IR and UV-vis spectroscopy, and molar conductivity measurements. Different *N*-donor amine skeletons including tridentate: L^1^ = [(2-pyridyl)-2-ethyl)-(3,4-dimethoxy)-2-methylpyridyl]methylamine and L^2^ = [(2-pyridyl)-2-ethyl)-(3,5-dimethyl-4-methoxy)-2-methyl-pyridyl]methylamine, and tetradentate: L^3^ = bis(2-ethyl-di(3,5-dimethyl-1*H*-pyrazol-1-yl)-[2-(3,4-dimethoxy-pyridylmethyl)]amine, tedmpza = tris[(2-(3,5-dimethyl-1*H*-pyrazol-1-yl)ethyl]amine and isp_3_tren = tris[(2-isopropylamino)ethyl)]amine ligands were employed. Molecular structural parameters such as nature of coligand, its chelate ring size and steric environment incorporated into its skeleton, which lead to adopting one of the two limiting geometries in these complexes and other reported compounds are analyzed and correlated to their assigned geometries in solutions. Similar analysis were extended to other five-coordinated halido-Cu(II) complexes.

## 1. Introduction

Copper is an essential element for life as it is incorporated into a variety of proteins and metalloenzymes, which occur in animals and plants and required to perform essential metabolic functions. Many of biologically active copper enzymes are utilized for electron transfer reactions, oxygen transport and oxygenation reactions [1,2]. The deficiency in copper or its high concentration in human tissues may lead to Menkes or Wilson diseases, respectively [3]. Over the last decade, a wide range of copper(II) complexes were demonstrated to be potentially effective as anticancer agents [4,5,6,7,8] and less toxic compared to the currently used platinum-based anticancer drugs [9]. Many of the designed anticancer Cu(II) are coordination compounds containing polyamines as coligands and chloride or bromide groups [5,6,7,8,10,11,12]. In some cases, these halido compounds are unstable in solutions and may undergo solvolysis or aquation with possible stereochemical geometry change [5,7,11,12].

The interaction of pseudohalides (isothiocyanate, NCS^−^; azide, N_3_^−^; and dicyanamide, dca) with Cu(II) salts in the presence of coligands resulted in the construction of polynuclear compounds with different dimensionality and nuclearity, and coordination polymers (CPs) with diverse topology [13,14,15,16,17,18,19]. This was attributed to the high ability of the pseudohalide ions to act as bridging ligands in a variety of bonding modes and mediating the magnetic interaction between Cu^2+^ centers [17,18,19,20], where efficient magnetic transmitting was found in case of the azide bridging compounds [17,19,20]. However, mononuclear pseudohalido-Cu(II) complexes are known to be formed too [21,22,23,24,25,26,27,28,29,30] and are very stable in aqueous and common organic solvents [27,28]. The Cu(II) centers in these compounds display variable geometries ranging from four—(square planar and tetrahedral) to six—(octahedral) as well as five-coordinated species. The latter species exist in solutions in equilibrium between the square pyramidal (SP) and trigonal bipyramidal (TBP) geometries as the energy barrier between the two species is very small [31]. Several structural parameters such as steric effect, nature of the chelate ring size (ligand bite) and skeleton of the coordinated coligand were proposed to account for the presence of specific predominant stereochemical geometry [22,23,24]. Taking into considerations the stability of the pseudohalido- vs. instability of some halido-copper(II) complexes, it is safe to point out that molecular structures do not always provide information about how these halido complexes exist in solution, especially if the halido-Cu(II) compounds persist in solutions [5,7].

**Scheme 1:**
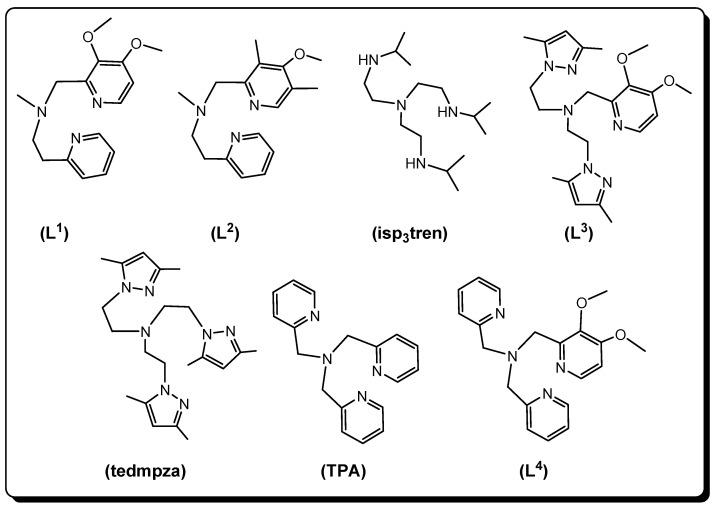
Structure formulas of some *N*-donor ligands used in this study. Tris(2-pyridylmethyl)amine (TPA) was illustrated in the scheme for comparison with other derivatives of the compound.

Thus, in order to investigate the above parameters that influence the geometry of the five-coordinated Cu(II) species, a novel series of mononuclear pseudohalide complexes derived from *N*-donors tri- and tetra-dentate amines with different coligand skeletons were synthesized. These include: [Cu(L^1^)(NCS)_2_] (**1**), [Cu(L^2^)(NCS)_2_] (**2**), [Cu(isp_3_tren)(N_3_)]ClO_4_ (**3**), [Cu(isp_3_tren)(dca)]ClO_4_ (**4**), [Cu(L^3^)(NCS)]ClO_4_ (**5**), [Cu(tedmpza)(dca)]ClO_4_·0.67H_2_O (**6**) and [Cu(L^4^)(dca)](ClO_4_)∙2H_2_O (**7**) complexes, where L^1^ = [(2-pyridyl)-2-ethyl)-(3,4-dimethoxy)-2-methylpyridyl]methylamine, L^2^ = [(2-pyridyl)-2-ethyl)-(3,5-dimethyl-4-methoxy)-2-methylpyridyl]methylamine, L^3^ = bis(2-ethyl-di(3,5-dimethyl-1*H*-pyrazol-1-yl)-[2-(3,4-dimethoxypyridylmethyl)]amine, L^4^ = bis(2-methylpyridyl)-[2-(3,4-dimethoxypyridyl-methyl)]amine, isp_3_tren = tris[(2-isopropylamino)ethyl)]amine and tedmpza = tris[(2-(3,5-dimethyl-1*H*-pyrazol-1-yl)ethyl]amine (Scheme 1). The molecular structures of these complexes were determined and analyzed as function of the index distortion parameters τ and correlated to the assigned geometries, which were derived from their visible electronic spectra in solutions. The results are discussed and compared to other related pseudohalido as well as halido compounds in an attempt to predict the predominant five-coordinated geometry in solution and for how far one can rely on this geometrical assignment.

## 2. Results and Discussion

### 2.1. Synthetic Aspects

The interaction of methanolic solutions containing Cu(ClO_4_)_2_·6H_2_O or Cu(NO_3_)_2_·3H_2_O with the tridentate amines L^1^ or L^2^ (Scheme 1) in the presence of NH_4_NCS and the stoichiometric ratios 1:1:1 or 1:1:2 afforded the mononuclear diisothiocyanato complexes [Cu(L^1^)(NCS)_2_] (**1**) and [Cu(L^2^)(NCS)_2_] (**2**), respectively, whereas the reaction of Cu(ClO_4_)_2_·6H_2_O with the tripod tetraamine ligand, L^3^ resulted in the formation of [Cu(L^3^)(NCS)]ClO_4_ (**5**). Similarly, bluish green complexes [Cu(isp_3_tren)(dca)]ClO_4_ (**4**), [Cu(tedmpza)(dca)]ClO_4_·0.67H_2_O (**6**), and [Cu(L^4^)(dca)](ClO_4_)·2H_2_O (**7**) were obtained from the reactions of methanolic solutions containing Cu(ClO_4_)_2_·6H_2_O and the corresponding tripod ligands isp_3_tren, tedmpza and L^4^ (Scheme 1) respectively, with an aqueous solution of Nadca in a 1:1:1 or even 1:1:2 molar ratios. The green azido complex, [Cu(isp_3_tren)(N_3_)]ClO_4_ (**3**) was obtained using a similar procedure as that described for the dicyanamido complexes. The isolated complexes were produced in moderate to excellent yields (60–95%).

Single crystals suitable for X-ray analysis were obtained either from dilute aqueous-methanol solutions (**3**, **4**) or recrystallization (CH_3_CN: **1**, **2**; CH_3_OH: **6**; and H_2_O: **5**). Attempts made to get good crystals of complex **7** failed. All the complexes were characterized by elemental microanalyses, IR and UV-vis spectra and molar conductivity measurements (Λ_M_ in acetonitrile solutions) as well as X-ray single crystals crystallography for complexes **1**–**6**. The molar conductivity of the complexes **3**–**7** were found to be in the range 147–163 Ω^−1^·cm^2^·mol^−1^, which are in complete agreement with the predicted formulas as 1:1 electrolytic nature [32]. On the other hand, [Cu(L^1^)(NCS)_2_] (**1**) and [Cu(L^2^)(NCS)_2_] (**2**) complexes produced Λ_M_ values of 9 and 11 Ω^−1^·cm^2^·mol^−1^, respectively. These values are fully consistent with their non-electrolytic behavior [32,33]. The complexes are soluble in CH_3_CN and CH_3_OH and their solution electronic or molar conductivity, which were measured over four days did not show any sign of solvolysis, geometrical changes, nor degradation.

### 2.2. IR Spectra of the Complexes

The IR spectra of the isothiocyanato compounds **1**, **2** and **4** display a strong stretching vibration at 2055, 2054, and 2091 cm^−1^ respectively, corresponding to the ν_as_(C≡N) frequency. This is most likely consistent with *N*-bonded isothiocyanate, which in general shows ν_as_ < 2110 cm^−1^, whereas the corresponding *S*-bonded isothiocyanate bonding mode reveals the ν_as_(C≡N) at higher frequencies (>2110 cm^−1^) [23,24,34,35,36]. The azido complex **3** displays a single very strong absorption band at 2055 cm^−1^ due to the asymmetric stretching vibration, ν_as_(N_3_) of the coordinated mono-dentate azido group [18,26,29]. Three strong bands were detected over the frequency region 2350–2170 cm^−1^ for the dicyanamido complexes **4** and **6** being comparable to those observed in the free dicyanamide stretching frequencies [ν_as_ + ν_sym_(C≡N)] 2286 cm^−1^, ν_as_(C≡N) 2232 cm^−1^ and ν_sym_(C≡N) 2179 cm^−1^ [37] and indicating monodentate *N*-nitrile dca [27,28,29]. However, we should mention that the dicyanamido complex **7** displayed four strong bands for the coordinated dca at 2300, 2236, 2176, and 2137 cm^−1^. In addition the compounds **3**–**7** reveal a single strong band over the range 1100–1070 cm^−1^ attributable to the ν(Cl-O) stretching vibration of the perchlorate counter ion. The split of this band (1090 and 1067 cm^−1^) in **7** is probably due to the reduction of the ClO_4_^−^ symmetry from T_d_ to C_3υ_ or C_2υ_. The water of crystallization in [Cu(tedmpza)(dca)]ClO_4_·0.67H_2_O (**6**) and [Cu(L^4^)(dca)]ClO_4_·2H_2_O (**7**) was detected as a broad band at 3448 and 3435 cm^−1^ respectively, due to ν_as_(O-H) stretching frequency.

### 2.3. Description of the Structures

#### 2.3.1. [Cu(L^1^)(NCS)_2_] (**1**), [Cu(L^2^)(NCS)_2_] (**2**) and [Cu(L^3^)(NCS)]ClO_4_ (**5**)

**Figure 1:**
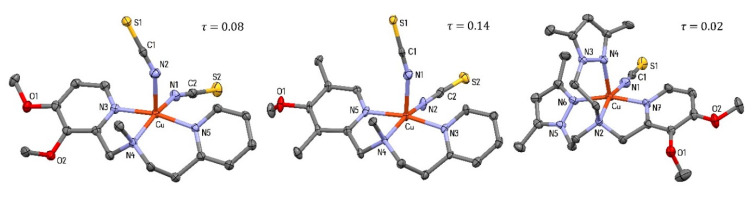
Perspective views of **1** (**left**), **2** (**center**) and complex cation of **5** (**right**). θ and ϕ bond angles (°) used for calculation of τ-values: **1**: N3-Cu-N5 167.20(3); N1-Cu-N4 162.13(9); **2**: N3-Cu-N5 168.4(3), N2-Cu-N4 160.3(3); and **5**: N6-Cu-N7 163.94(12), N1-Cu-N2 162.46(12).

The isothiocyanate compounds **1** and **2** form neutral monomeric complexes, whereas **5** crystallizes as a mononuclear complex cation together with perchlorate counter anion. In **1** and **2**, the Cu(II) centers are ligated by two terminal *N*-coordinated NCS^−^ anions and three donor *N*-atoms of the tri-dentate amine ligands L^1^ and L^2^, respectively, while in complex cation of **5**, the Cu(II) center is ligated by one terminal *N*-coordinated NCS^−^ anion and four donor *N*-atoms of the tetra-dentate amine ligand L^3^ (Figure 1). The CuN_5_ chromophores of the metal centers exhibit distorted and slightly elongated square pyramidal (SP) geometry with τ-values of 0.08 for **1**, 0.14 for **2**, and 0.02 for **5** [38]. In the neutral complexes the apical position is occupied by *N*-NCS^−^, with Cu-N apical bond distances of 2.188(2) and 2.165(7) Å, for **1** and **2**, respectively, whereas in **5** the apical position is occupied by N4 of the tetra amine L^3^ ligand [Cu-N4 = 2.232(3) Å]. The basal Cu-N bond lengths in the three compounds vary from 1.955(3) to 2.087(3) Å. The central N(amine) donor atoms of L^1^, L^2^, and L^3^ molecules, respectively, are placed trans to the basal *N*-(NCS) anions, which have the shortest Cu-N bonds [1.955(3)–1.988(6) Å]. The terminal NCS^−^ anions have the following bond parameters: N-C: 1.153(3)–1.163(10) Å; C-S: 1.626(4)–1.649 Å; Cu-N-C 149.0(2)–175.0(3)°; N-C-S: 177.9(7)–179.5(4)°.

#### 2.3.2. [Cu(isp_3_tren)(N_3_)]ClO_4_ (**3**), [Cu(isp_3_tren)(dca)]ClO_4_ (**4**), and [Cu(tedmpza)(dca)]ClO_4_∙0.67H_2_O (**6**)

**Figure 2:**
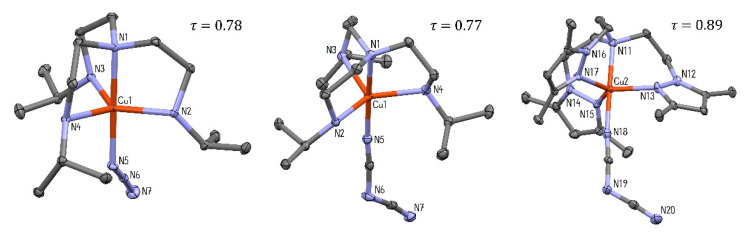
Perspective views of complex cations of **3** (**left**), **4** (**center**), and **6** (**right**). θ and ϕ bond (°) used for calculation of τ-values: **3**: N5-Cu1-N1 179.28(7); N2-Cu1-N3 132.54(6); **4**: N5-Cu1-N1 176.21(14), N2-Cu1-N4 129.85(14); **6**: N1-Cu1-N8 176.0(2), N3-Cu1-N7 128.2(2); N11-Cu2-N18 178.4(2), N15-Cu2-N17 125.3(2); and N21-Cu3-N28 176.7(2), N23-Cu3-N27 127.8(2).

The mononuclear [Cu(isp_3_tren)(X)]^+^ complex cations, [X = N_3_ for **3** and dca for **4**] co-crystallize with ClO_4_^−^ counter anions. Three crystallographic independent [Cu(tedmpza)(dca)]^+^ cations with three ClO_4_^−^ counter anions and two solvent water molecules are located in the triclinic unit cell of **6**. Each Cu(II) center in compounds **3**, **4**, and **6** is ligated by one terminal *N*-coordinated pseudohalide anion and four *N*-donor atoms of the tetra-dentate amine ligands (isp_3_tren or tedmpza) (Figure 2). The CuN_5_ chromophores of the copper centers exhibit slightly distorted trigonal bipyramidal (TBP) geometry with τ-values of 0.78 for **1**, 0.77 for **2**, and 0.78, 0.89, and 0.82 for Cu1-Cu3 of **6**, respectively [38]. The axial positions are occupied by one *N*-terminal pseudohalide anion and the central N(amine) donor atom of the tetra-dentate molecules, whereas the equatorial positions are occupied by three *N*-donor atoms of the arms of the isp_3_tren or tedmpza molecules. The shortest bond distances within each CuN_5_ polyhedron are observed for the Cu-N(N_3_) [1.9735(17) Å] and Cu-N(dca) [from 1.951(6) to 1.971(5) Å], while the axial Cu-N(amine) bond distances are in the range from 2.041(3) to 2.097(5) Å. The equatorial Cu-N bond lengths vary from 2.043(6) to 2.2417(17) Å. The terminal azide anion in **3** has N5-N6 and N6-N7 bond distances of 1.193(3) and 1.163(5) Å, and Cu-N5-N6 and N5-N6-N7 bond angles of 130.37(14) and 176.3(2)°, respectively. The terminal dca anions in **4** and **6** have the following bond parameters: C-N(nitrile): from 1.107(10) to 1.162(8) Å; C-N(amine): from 1.290(9) to 1.339(11) Å; Cu-N-C: from 162.5(7) to 173.6(6)°; N-C-N: from 163.5(9) to 175.9(8)°, and C-N-C: from 116.1(8) to 122.1(4)°.

In **6**, the three complex cations differ mainly in the torsion around the dca ligation [torsion angles: Cu1-N8···N9···N10 = −116.87°; Cu2-N18···N19···N20 = 47.37°; Cu3-N28···N29···N30 = 104.94°]. Furthermore, only dca donor atom N30 of complex cation of Cu3 is involved in a hydrogen bond of type O-H···N to water molecule O13, which forms also a hydrogen bond of type O-H···O to perchlorate counter anion of Cl2. The second water molecule O14 forms only hydrogen bonds of type O-H···O to water molecule O13 and perchlorate counter anion of Cl3 (see packing plot Figure S6, and Table S4, Supplementary Materials).

### 2.4. UV-Vis Spectra of the Complexes

The acetonitrile electronic spectra of the Cu(II) complexes **1**–**7** can be classified into two categories:

(1) Complexes **1** and **2**, which were derived from tridentate ligands L^1^ and L^2^ exhibit a single broad absorption around 676 nm.

(2) Complexes **3**–**7** revealed a shoulder or relatively weak band over the region 630–740 nm and a broad band over the wavelength region 840–940 nm.

In addition, the very strong intense band located around 390 nm for compounds **1**, **2**, **5** and **6**, and at 350 nm in **7** can be assigned to ligand-metal charge transfer transition (LMCT). The visible spectral features observed in the two categories of complexes are consistent with five-coordinated Cu(II) geometries. In general, Cu^2+^ in square pyramidal geometry (SP) exhibits broad band in the visible region (590–780 nm), which occasionally may or may not be associated with a low-energy shoulder at λ > 800 nm (spin forbidden), whereas trigonal bipyramidal (TBP) are known to display a single *d-d* band at λ > 800 nm with a high-energy shoulder in the visible region [39]. Therefore, based on this criterion, the acetonitrile visible spectral data of the complexes under investigation are consistent with SP geometry in complexes **1** and **2** [25,40], whereas TBP environment seems to be dominating in the second category of complexes **3**–**7** coordinated to tripodal tetraamine coligands (L^3^, L^4^, isp_3_tren, tedmpza; Scheme 1) [41]. It is important to point out that the intensity of band at λ > 800 nm in TBP geometries should be higher than the shoulder or the band located at λ < 800 nm (small intense maxima). However, it should be emphasized that in most cases, the complexes may exhibit intermediate geometries which are slightly distorted from the ideal SP or TBP.

### 2.5. Molecular vs. Solution Structures in Five-Coordinated Cu(II) Complexes

**Figure 3:**
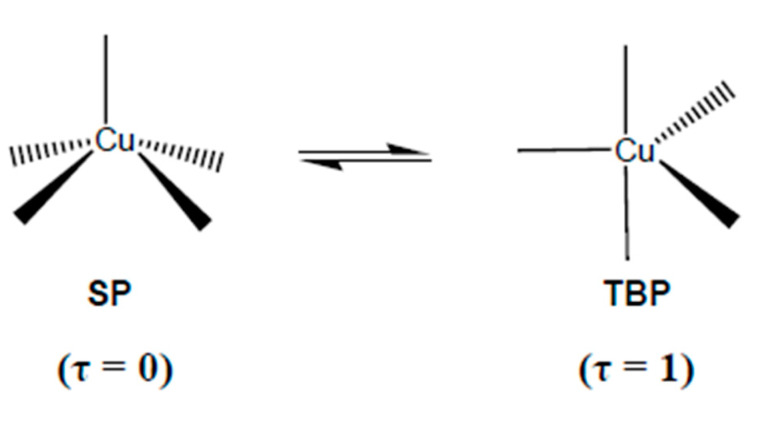
Topological transformation between square pyramidal (SP) and trigonal bipyramidal (TBP) geometries.

Five-coordinated metal(II) complexes, especially when the central metal ion is Cu(II), Co(II), or Zn(II) are quite known to crystallize in two stereochemical environments around the central M^2+^ ion namely square pyramidal (SP) and trigonal bipyramidal (TBP) (Figure 3) because the energy barrier between the two possible limiting geometries is rather small [32]. Several factors were considered to contribute to this phenomenon such as the nature of the central metal ion, chelate ring size, and coligand’s skeleton as well as the steric substituent effect incorporated into its structure. These factors play crucial roles in adopting one of the two geometries. From the molecular structure point of view, it is possible to quantify the extent of distortion of the metal polyhedron through the evaluation of the structural index parameter (τ), which is defined as the ratio between the two basal angles θ and ϕ in the given five-coordinated complex, τ = (θ − ϕ)/60, where θ ≥ ϕ. A perfect TBP is associated with ϕ = 120° and θ = 180°, leads to τ = 1, whereas in an ideal SP geometry θ = ϕ = 180° leading to τ = 0 [38]. In real SP structures, the metal ion is always located out of the equatorial plane towards the axial bond and the resulting C_4υ_ geometry is still be characterized by τ ≈ 0 (θ ≈ ϕ < 180°). In the solid state, one of the two conformations is isolated as a predominant geometrical isomer. However, in solutions the situation is different and it is not necessary that the molecular structure, which isolated persists in solution.

This section is devoted to consider the molecular and solution structures of the mononuclear pseudohalide-Cu(II) five-coordinated complexes **1**–**6** together with some other related compounds, which are also mononuclear and their structures were determined in both the solid and solution states. This comparison will allow us to analyze the structural parameters leading to the isolation of a specific geometrical conformation (TBP vs. SP). Therefore, a number of these compounds were compiled in Table 1 together with their corresponding τ values. Furthermore, the visible spectra of the same series of compounds in solutions are collected in Table 2. Comparing the molecular and solution structures should provide some information about the validity of the predicted geometry in solutions and to what extent one can rely on the structural results in solutions.

Inspection of the data given in Table 1 reveals that five-coordinated isothiocyanato-Cu(II) complexes **1**, **2** and **8**–**20**, which were derived from linear tridentate, tripodal tetra- or cyclic-tetradentate (cyclen-tmpa) ligands show very high tendency to adopt SP geometry regardless the chelate ring sizes (five or six). In contrast, the corresponding azido **3**, **25**, and **27**, and the dicyanamido series **4**, **8**, and **36** constructed from tripodal tetradentate ligands with all compounds displaying five-membered chelate rings (tren, Me_3_tren, isp_3_tren, TPA) indicate more preference for TBP geometry. This stereochemical structure changes to SP as the number of six-membered rings increases as this was the case on going from TPA (complexes **27** and **36** with τ values of 0.91 and 0.95, respectively) to pmap or tepa in compounds **30** and **37** as well as in [Cu(tedmpza)(N_3_)]ClO_4_ (**31**) or from tren to trpn in complexes **35** and **32**, respectively. Most likely this is attributed to the flexibility of the six-membered chelate rings. Similar SP geometry was also observed when steric effect at the coordinated amines are fully alkylated as in [Cu(pmedien)(dca)_2_] (**33**). Moreover, moderate substituents into the pyridyl groups of TPA, especially at position 6 results in increasing the distortion of the TBP geometry observed in TPA complexes (see complexes **28**: τ = 0.65 and **29**: τ = 0.65 and 0.73; 0.68; 0.50; 0.61; 0.31, [47]). However, we should mention that some pronounced deviations were found in the azido compound **31** and its corresponding dicyanamido, [Cu(tedmpza)(dca)]ClO_4_·0.67H_2_O (**6**) and in related tris pyrazolyl, [Cu(tepza)(dca)]ClO_4_ (**38**) (based on the above analysis both are expected to exhibit SP). These deviations may result from the crystal packing.

Transferring the compound of which its molecular structure was determined into solution is a complicated issue due to possible solvolysis, stereochemical change or decomposition of the complex. The question now which class of compounds can retain their geometries in solutions? To provide a satisfactory answer to this question, the solution visible electronic spectra of the compounds shown in Table 1 are collected in Table 2 with predicted predominant geometries based on the analysis provided in Section 2.4. Probably, we should mention that the complexes **1**–**7** were shown to be very stable in common organic solvents for several days. Similar behavior was also reported in complexes [Cu(trpn)(N_3_)]ClO_4_ (**32**), [Cu(tepa)(dca)]ClO_4_ (**37**), and [Cu(tren)(dca)]ClO_4_ (**35**) [27,28,48].

Inspection of the data in Table 2, and the corresponding molecular structures given in Table 1, surprisingly indicate complete agreement between the predicted geometries in solution and the molecular structures. This finding means we can rely with confidence that the determined molecular structures in five-coordinated pseudohalido-Cu(II) complexes are retained in solutions without any stereochemical change.

Since many of the halido-Cu(II) complexes are now extensively used to mimic biological systems, especially those derived from tripodal and linear tetraamine coligands (N4), [Cu(N4)X]^+^ in DNA cleavage and the cytotoxicity studies as potential anticancer agents [4,5,6,7,8,49,50,51,52], it is worthwhile to shed the light on the behavior of this class of compounds in solutions in order check the validity of geometrical structures at the two levels. The data compiled in Table 3 reveals that increasing the steric crowding at the TPA through successive methylation at the 6th position of the pyridyl groups resulted in the conversion from perfect TBP geometry as in [Cu(TPA)Cl]^+^ to distorted SP in [Cu(6-MeTPA)Cl]^+^ and [Cu(6-Me_2_TPA)Cl]^+^, and to intermediate geometry in the most sterically hindered [Cu(6-Me_3_TPA)Cl]^+^ complex ion (Table 3: compounds **42**–**46**), a result which was unexpected. Probably, this behavior was enhanced when more substituents were incorporated into the TPA-pyridyl group as this was seen in complexes **55**–**58**, where the TBP is becoming more stabilized [8]. The same trend was observed in the quinolyl derivatives of TPA: [Cu(BPQA)Cl]^+^ and [Cu(BQPA)Cl]^+^, where geometry changes to dist. SP and then to dist. TBP in the latter case (compounds **51**–**54**). Moreover, SP geometries seem to be the most preferable species with bidentate (L^9^), linear tri-(L^8^) and tetra-dentate (L^10^) ligands as these were the cases of complexes **61**–**63** [49,50,51].

On the other hand, Cu(II) complex ions with only five-membered chelate ring size adopt almost perfect TBP geometry as it was demonstrated in [Cu(TPA)X]^+^ (X = Cl, F) (**39**–**41**) and [Cu(Me_6_tren)X]^+^ (X = Cl, Br); (**59**, **60**) [52,53,54,58] and dramatic shift to almost perfect SP was observed when six-membered chelate rings were employed, [Cu(N4)Cl]^+^ (N4 = pmea, pmap, tepa: **48**–**51**) [56,57]. This trend is similar and more pronounced in this series compared to those observed above in the corresponding pseudohalide species.

Although data compiled in Table 3 for the assigned geometry in solutions shows that the agreement of the molecular and solution structures is not bad in most cases, results must be considered with caution due to possible geometrical changes in some cases such as in [Cu(6-MeTPA)Cl]^+^ (**42** and **43**) and [Cu(BPQA)Cl]^+^ (**51** and **52**) [8,55]. In addition, it has been reported that some (Cu(II) complexes are unstable in organic solvents or aqueous media and may undergo solvolysis/aquation as this was evidenced in complex [Cu(L^9^)Cl_2_] (**61**). The conductance measurements of this compound showed that it is non-conducting in dry DMF, DMSO, and CH_3_CN as this was expected from its molecular formula but in MeOH and aqueous/DMF solutions, the complex displayed both 1:1 and 1:2 electrolytic behaviors, respectively indicating the loss of chloride ligand(s) and the formation of [Cu(L^9^)Cl(H_2_O)]^+^ and [M(L^9^)(H_2_O)_2_]^2+^ species [49]. Similar behaviour was also observed in [Cu(L^10^)(H_2_O)(Cl)_2_] (**62**) and [Cu(L^11^)(Br)_2_] (**63**) complexes [50,51].

## 3. Materials and Methods

### 3.1. Materials and Physical Measurements

*N*-Methyl-*N*-(2-pyridyl)ethyl)amine was purchased from Maybridge, Belgium and tris[(2-isoptopylamino)ethyl)]amine from Aldrich. 3,4-Dimethoxy-2-chloromethylpyridine, 3,5-dimethyl-4-methoxy)-2-chloromethylpyridine, bis(2-methylpyridyl)amine, bis(2-chloroethyl)-amine and 3,5-dimethylpyrazole were purchased from TCI-America (Portland, OR, USA). All other materials were reagent grade quality. Infrared spectra were recorded on a Cary 630 (ATR) spectrometer (Santa Clara, CA, USA) or JASCO FT/IR-480 plus spectrometer (Oklahoma City, OK, USA) using KBr pellets. Electronic spectra were recorded using Agilent 8453 HP diode UV-vis Spectrophotometer (Santa Clara, CA, USA). The molar conductivity of a solution sample was determined from Λ_M_ = (1.0 × 10^3^ κ)/[Cu], where κ is the specific conductance and [Cu] is the molar concentration of the complex. The measurements were performed using Mettler Toledo Seven Easy conductometer (Columbus, OH, USA) and calibration was determined by the aid of 1413 μS/cm conductivity standard. Elemental microanalyses were carried out by Atlantic Microlaboratory (Norcross, GA, USA).

**Caution**! Salts of perchlorate and azide as well as their metal complexes are potentially explosive and should be handled with great care and in small quantities.

### 3.2. Synthesis of the Compounds

*Synthesis of the organic ligands*: The compounds [(2-pyridyl)-2-ethyl)-(3,4-dimethoxy)-2-methylpyridyl]methylamine (L^1^) and [(2-pyridyl)-2-ethyl)-(3,4-dimethoxy)-2-methylpyridyl]-methylamine (L^2^) [59], [bis(3,5-dimethyl-ethyl-pyrazol-1*H*-1-yl)-(3,4-dimethoxy-2-methylpyridyl)]amine (L^3^) [60], [bis(2-methylpyridyl)-(3,4-dimethoxy-2-methylpyridyl)]-amine, (L^4^) [8] and tris(3,5-dimethyl-ethyl-1*H*-pyrazol-1-yl)amine (tedmpza) [61,62] were prepared and characterized according to the published procedures.

*Synthesis of [Cu(L^1^)(NCS)_2_]* (**1**): To a mixture containing [(2-pyridyl)-2-ethyl)-(3,4-dimethoxy)-2-methylpyridyl]methylamine, L^1^ (0.145 g, 0.50 mmol) and Cu(ClO_4_)_2_·6H_2_O (0.190 g, 0.5 mmol) or Cu(NO_3_)_2_·3H_2_O (0.122 g, 0.50 mmol) dissolved in MeOH (20 mL) NH_4_NCS (0.076 g, 1.0 mmol) was added. The resulting green solution was heated for 5 min, filtered through celite and then allowed to stand at room temperature. After four days, the precipitate formed was collected by filtration and recrystallized from CH_3_CN to afford green single crystals suitable for X-ray analysis (yield: 0.215 g, 92%). Characterization: Anal. Calcd. for C_18_H_21_CuN_5_O_2_S_2_ (467.06 g/mol): C, 46.29; H, 4.53; N, 14.99. Found: C, 46.49; H, 4.47; N, 15.00%. Selected IR bands (ATR, cm^−1^): 2997 (vw), 2931 (vw), 2879 (vw), 2835 (vw) ν(C-H); 2055 (vs) ν_as_(N≡C) (NCS^−^); 1608 (s), 1496 (s), 1444 (s), 1362 (m), 1304 (s), 1231 (s), 1172 (m), 1069 (s), 1000 (s), 902 (m), 820 (s), 787 (m), 769 (vs), 733 (s). UV-vis (CH_3_CN), λ_max_, nm (ε_max_, cm^−1^ M^−1^): 385 (1180), 676 (190). Λ_M_ (CH_3_CN) = 9 Ω^−1^·cm^2^·mol^−1^.

*Synthesis of [Cu(L^2^)(NCS)_2_]* (**2**): To a mixture containing [(2-pyridyl)-2-ethyl)-(3,5-dimethyl-4-methoxy)-2-methylpyridyl]methylamine, L^2^ (0.140 g, 0.50 mmol) and Cu(ClO_4_)_2_·6H_2_O (0.190 g, 0.5 mmol) dissolved in MeOH (20 mL) NH_4_NCS (0.076 g, 1.0 mmol) was added. The resulting green solution was heated for 5 min, filtered and allowed to crystallize at room temperature. After two days, the produced precipitate was collected by filtration and recrystallized from CH_3_CN to afford green single crystals suitable for X-ray analysis (yield: 0.178 g, 77%). Characterization: Anal. Calc. for C_19_H_23_CuN_5_OS_2_ (465.09 g/mol): C, 49.07; H, 4.08; N, 15.06. Found: C, 49.26; H, 4.99; N, 15.13%. Selected IR bands (KBr, cm^−1^): 3003 (vw), 2919 (vw), 2872 (vw), ν(C-H); 2054 (vs) ν_as_(N≡C) (NCS^−^); 1606 (m), 1489 (m), 1475 (m), 1442 (m), 1268 (m), 1110 (m), 1012 (m), 999 (m), 904 (w), 880 (w), 769 (vs). UV-vis (CH_3_CN): λ_max_, nm (ε_max_, cm^−1^ M^−1^): 388 (1214), 676 (196). Λ_M_ (CH_3_CN) = 11 Ω^−1^·cm^2^·mol^−1^.

*Synthesis of [Cu(isp_3_tren)(N_3_)]ClO_4_* (**3**): To a mixture containing of Cu(ClO_4_)_2_·6H_2_O (0.190 g, 0.5 mmol) and tris(2-isopropylamino)ethyl)amine dissolved in 10 mL MeOH), an aqueous solution of NaN_3_ (0.033 g, 0.5 mmol in 5 mL H_2_O) was added dropwise. The resulting green solution was heated for 5 min, filtered through celite and allowed to stand at room temperature. After 3 days, the shiny green crystals which separated were collected by filtration, washed with propan-2-ol, ether and dried in air (overall yield: 0.210 g, 88%). Characterization: Anal. Calcd. for C_15_H_36_ClCuN_7_O_4_ (MM = 477.49 g/mol): C, 37.73; H, 7.60; N, 20.53%. Found: C, 37.80; H, 7.69; N, 20.64%. Selected IR bands (ATR, cm^−1^): 3269 (m), 3215 (m) ν(N-H); 2974 (w), 2879 (w) ν(C-H); 2055 (vs) ν_as_(N_3_); 1067 (vs) ν(Cl-O) (ClO_4_^−^). UV-vis in CH_3_CN: λ_max_ in nm (ε_max_, M^−1^cm^−1^): 695 (364, vbr), 870 (397, b). Λ_M_ (CH_3_CN) = 157 Ω^−1^·cm^2^·mol^−1^.

*Synthesis of [Cu(isp_3_tren)(dca)]ClO_4_* (**4**): The complex was isolated as tiny blue single crystals after two days using a procedure similar to that described for complex **3**, except Nadca was used instead of sodium azide (overall yield: 0.196 g, 78%). Characterization: Anal. Calcd. for C_17_H_36_ClCuN_7_O_4_ (MM = 501.51 g/mol): C, 40.71; H, 7.24; N, 19.55%. Found: C, 40.04; H, 7.32; N, 19.76%. Selected IR bands (ATR, cm^−1^): 3263 (w), 3228 (m) ν(N-H); 2988 (m), 2942 (m), 2885 (m) ν(C-H); 2301 (s), 2238 (s), 2172 (vs) ν_as_(N≡C) (dca); 1100 (vs, b) ν(Cl-O) (ClO_4_^−^). UV-vis in CH_3_CN: λ_max_ in nm (ε_max_, M^−1^cm^−1^): ~660 (sh), 837 (397, br). Λ_M_ (CH_3_CN) = 163 Ω^−1^·cm^2^·mol^−1^.

*Synthesis of [Cu(L^3^)(NCS)]ClO_4_* (**5**): To a mixture containing Cu(ClO_4_)_2_·6H_2_O (0.190 g, 0.50 mmol) and [bis(3,5-dimethyl-ethyl-pyrazol-1*H*-1-yl)-(3,4-dimethoxy-2-methylpyridyl)]amine, L^3^ (0.206 g, 0.50 mmol) dissolved in MeOH (20 mL), NH_4_NCS (0.038 g, 0.50 mmol) was added. The dark green solution was heated to boiling and filtered while hot through celite. After 2 h, the resulting green precipitate, which separated was collected by filtration, washed with propan-2-ol, ether and dried in air (yield: 0.30 g, 95%). Recrystallization of the product from H_2_O afforded medium spring green single crystals suitable for X-ray analysis. Characterization: Anal. Calcd. for C_23_H_32_ClCuN_7_O_6_S (MM = 633.61 g/mol): C, 43.60; H, 5.09; N, 15.47%. Found: C, 43.81; H, 5.06; N, 15.65%. Selected IR bands (ATR, cm^−1^): 3121 (w), 3089 (w), 2935 (w), 2844 (w) ν(C-H); 2091 (s) ν_as_(N≡C) (NCS^−^); 1604 (m), 1552 (m), 1502 (m), 1438 (m), 1309 (s), 1236 (w); 1073 (vs) ν(Cl-O) (ClO_4_^−^). UV-vis (CH_3_CN): λ_max_ in nm (ε_max_, M^−1^cm^−1^): 387 (1020), ~630 (sh), ~940 (76). Λ_M_ (CH_3_CN) = 149 Ω^−1^·cm^2^·mol^−1^.

*Synthesis of [Cu(tedmpza)(dca)]ClO_4_·0.*67H_2_O (**6**): A mixture of Cu(ClO_4_)_2_·6H_2_O (0.190 g, 0.50 mmol) and tris[(3,5-dimethy-1*H*-pyrazol-yl)ethyl]amine (tedmpza) dissolved in MeOH (20 mL) was treated with an aqueous solution containing Nadca (0.045 g, 0.50 mmol in 10 mL H_2_O). This was followed by heating for 10 min, filtration through celite and the solution was allowed to stand at room temperature. The bluish green crystalline compound, which was isolated after four days (yield: 0.185 g, 60%) was recrystallized from CH_3_OH to afford shiny bluish green single crystals suitable for X-ray analysis. Characterization: Anal. Calcd. for C_23_H_34.34_ClCuN_10_O_4.67_ (MM = 624.64 g/mol): C, 44.22; H, 5.54; N, 22.42%. Found: C, 44.42; H, 5.56; N, 22.48%. Selected IR bands (ATR, cm^−1^): 3448 (w) ν(O-H); 3137 (vw), 2926 (w) ν(C-H); 2347 (w), 2270 (w), 2203 (s) ν_as_(N-C) (dca); 1629 (w), 1552 (s), 1465 (m), 1424 (m), 1390 (m), 1331 (w) (pyrazolyl ring); 1091 (vs) ν(Cl-O) (ClO_4_^−^). UV-vis in CH_3_CN: λ_max_ in nm (ε_max_, M^−1^cm^−1^): 387 (960), ~730 (90, sh), ~940 (110, br). Λ_M_ (CH_3_CN) = 147 Ω^−1^·cm^2^·mol^−1^.

*Synthesis of [Cu(L^4^)(dca)](ClO_4_)·2H_2_O* (**7**): This was synthesized as described above for **6** except [bis(2-methylpyridyl)-(3,4-dimethoxy-2-methylpyridyl)]amine, L^4^ was used instead of tedmpza. The complex was isolated as bluish green crystalline compound after 2 days (overall yield: 0.220 g, 71%). Characterization: Anal. Calcd. for C_22_H_26_ClCuN_7_O_8_ (MM = 615.48 g/mol): C, 42.93; H, 4.26; N, 15.93%. Found: C, 42.80; H, 4.14; N, 15.93%. Selected IR bands (ATR, cm^−1^): 3435 (s,b), ν(O-H); 3076 (w), 2946 (w), 2848 (w) ν(C-H); 2300 (m), 2236 (s), 2176 (vs), 2137 (vs) ν_as_(N≡C) (dca); 1606 (s), 1501 (m), 1439 (m), 1366 (m), 1308 (m); 1090 (vs), 1067 (vs) ν(O-Cl) (ClO_4_^−^); 843 (w), 769 (m), 625 (m). UV-vis in CH_3_CN: λ_max_ in nm (ε_max_, M^−1^cm^−1^): 350 (923), ~665 (sh), 880 (256, br). Λ_M_ (CH_3_CN) = 148 Ω^−1^·cm^2^·mol^−1^.

### 3.3. X-ray Crystal Structure Analysis

Suitable single crystals of **1**–**6** were carefully selected under polarizing microscope and mounted on a goniometer head. A Bruker-AXS APEX II CCD diffractometer (Karlsruhe, Germany) operating with Mo-Kα radiation (λ = 0.71073 Å) at 100(2) K was used for single crystal data collection. In Table 4 relevant crystallographic data together with some features of the structure refinements are summarized. APEX and the SADABS computer programs [63,64,65] were used for data processing, LP and absorption corrections. The SHELX [66,67] program library was used for structure solution (direct methods) and refinement (full-matrix least-squares methods with F^2^ data). Non-hydrogen atoms were refined with anisotropic displacement parameters, while the hydrogen atoms were located from difference Fourier maps, and included in the refinements with isotropic displacement factors. HFIX geometrical constraints were applied for H atoms with parent C atoms. Additional computer programs: Mercury and PLATON [68,69].

The structure **3** crystallizes in chiral space group P2_1_2_1_2_1_ includes only a single ligand enantiomer (R at each chiral N) and Flack parameter is 0.022(8). In structure of **5** a void centered at position 0.393, 0.568, 0.914 is separated by 3.22 Å from O2 acceptor atom. Its size of 37 Å^3^ corresponds to volume of one water molecule. No SQUEEZE treatment was applied. In case of **4** low but still acceptable crystal quality causes R_int_ of 0.141 and poor data/parameter ratio of 9.94.

## 4. Conclusions

Two series of geometrical five-coordinated pseudohalido-Cu(II) complexes were separated and structurally characterized: distorted square pyramidal: [Cu(L^1^)(NCS)_2_] (**1**), [Cu(L^2^)(NCS)_2_] (**2**), and [Cu(L^3^)(NCS)]ClO_4_ (**5**), and distorted trigonal bipyramidal: [Cu(isp_3_tren)(N_3_)]ClO_4_ (**3**), [Cu(isp_3_tren)(dca)]ClO_4_ (**4**), and [Cu(tedmpza)(dca)]ClO_4_∙0.67H_2_O (**6**). The geometrical identities of the complexes were also determined in solution and results are compared to other related reported compounds as well as to those derived from halido-Cu complexes with similar stereochemistry. In the pseudohalido complexes the consistency between the molecular and the assigned structures in solution was excellent (Table 1 and Table 2) indicating we can rely with confidence that the molecular geometrical structure, which was determined in the solid state is retained in solution. On the other hand, in the five-coordinated halido-Cu(II) complexes the picture was a little bit complicated as it was turned out that the molecular structure geometries, sometimes do not persist in solutions and may stereochemically change and/or hydrolyzed as this was observed in [Cu(6-MeTPA)Cl]^+^ (Table 3: **42**, **43**) and [Cu(BPQA)Cl]^+^ (**51** and **52**) [8,55] and in the dist. SP series [Cu(L^9^)Cl_2_] (**61**), [Cu(L^10^)(H_2_O)(Cl)_2_] (**62**), and [Cu(L^11^)(Br)_2_] (**63**) [49,50,51]. This result is important because five-coordinated halido-Cu(II) complexes, which are constructed from *N*-donor coligands are widely used as potential anticancer agents and to mimic biological systems [3,4,5,6,7,8,44,49,50,51,52] and under these conditions the geometry and possibly the identity of the reactive species could be different from the originally isolated solid [5,6,7,8,44,49,50,51,52]. In these cases, results in solutions must be handled with great care and we cannot rely with confidence on molecular structure determination to assign the structures in solutions.

To account for why a stereospecific geometry is adopted by a given five-coordinated Cu(II) compound (TBP vs. SP), number of parameters including chelate ring size, nature and steric environment imposed in the coligand’s skeleton were addressed [22,23,24]. In general, TBP structures are dominating in the solid state when the central Cu^2+^ ion is coordinated to tripod tetradentate ligands containing only five-membered chelate rings (Table 1 and Table 3: **3**, **4**, **20**–**22**, **36**, **37**, **39**–**41**, **54**, **56**–**60**) [8,27,28,29,41,52,53,54]. Also, the tendency for stabilization of SP geometry is enhanced (**18**, **30**–**32**, **47**–**50**) [23,30,43,48,56,57] as the chelate ring sizes are increased to six-membered through insertion of CH_2_ group(s) into the ethylenic tripod arms. Interestingly, this analysis was proved to be universal in other five-coordinated complex ions such [Cu(tren)(NH_3_)]^2+^, [Cu(Me_3_tren)(NCCH_3_)]^2+^, [Cu(TPA)(H_2_O)]^2+^ and [Cu(233)(NH_3_)]^2+^ (233 = *N*-(2-aminoethyl)-*N*,*N*-bis(3-aminopropyl)amine), where the first three complexes were displaying distorted TBP geometry, whereas the latter one exhibits distorted SP geometry [55,70,71,72]. The tendency for SP structures was also observed when CH_3_ groups were introduced at the 6th positions of the pyridyl arms in TPA or when one of the pyridyl groups was replaced with quinolyl groups (**42**, **43**, **45**, **52**) [8,56]. Distorted SP environment was clearly dominating in all complexes derived from tridentate ligands regardless the chelate ring sizes (**1**, **2**, **8**–**16**, **30**) [22,23,24,40,42,43], the macrocyclic complex [Cu(cyclen-tpam)(NCS)]ClO_4_·3H_2_O (**19**) [23] as well as in [Cu(Medpt)(ONO)(H_2_O)]^+^ (τ = 0.03) and [Cu(dien)(ONO)]^+^ (τ = 0.13), where Medpt = 3,3′-diamino-*N*-methyldipropylamine and dien = diethylenetriamine [73]. However, still some few contradictions exist in the geometrical behaviors of the SP [Cu(tedmpza)(N_3_)]ClO_4_ (**31**) [23] and its corresponding distorted TBP structure [Cu(tedmpza)(dca)]ClO_4_·0.67H_2_O (**6**), which was expected to display SP geometry based on the previous analysis as well as in [Cu(tepza)(NCS)]ClO_4_ (**17**), which exhibits intermediate geometry and the TBP [Cu(tepza)(dca)]ClO_4_ (**38**) [25], where both were predicted to display distorted SP geometry.

## Figures and Tables

**Table 1:** Geometry and distortion parameter (τ) in mononuclear five-coordinated pseudohalido-copper(II) complexes derived from *N*-donor ligands in the solid states ^a^.

Complex		τ Value	Geometry ^b^	Ref.
[Cu(L^1^)(NCS)_2_]	(**1**)	0.08	dist. SP	This work
[Cu(L^2^)(NCS)_2_]	(**2**)	0.14	dist. SP	This work
[Cu(bdmpzpy)(NCS)_2_]	(**8**)	0.25	dist. SP	[23]
[Cu(Mebedmpza)(NCS)_2_]	(**9**)	0.06	dist. SP	[22]
[Cu(Bzbedmpza)(NCS)_2_]	(**10**)	0.26	dist. SP	[22]
[Cu(DPA)(NCS)(ClO_4_)]	(**11**)	0.21	dist. SP	[23]
[Cu(mpipy)(NCS)_2_]	(**12**)	0.11	dist. SP	[42]
[Cu(mpiq)(NCS)_2_]	(**13**)	0.33	dist. SP	[42]
[Cu(pzdepy)(NCS)]ClO_4_	(**14**)	0.11	dist. SP	[43]
[Cu(Me_3_dpt)(NCS)_2_] ^c^	(**15**)	0.37, 0.49	dist. SP, intermed.	[40]
[Cu(bedmpza)(NCS)_2_] ^c^	(**16**)	0.24, 0.46	dist. SP, intermed.	[24]
[Cu(tepza)(NCS)]ClO_4_	(**17**)	0.47	Intermed.	[25]
[Cu(bdmpe)(NCS)]ClO_4_	(**18**)	0.17	dist. SP	[30]
[Cu(L^3^)(NCS)]ClO_4_	(**5**)	0.02	SP	This work
[Cu(cyclen-tpam)(NCS)]ClO_4_·3H_2_O	(**19**)	0.00	SP	[23]
[Cu(L^5^)(NCS)]ClO_4_	(**20**)	0.43	dist. SP	[44]
[Cu(L^5^)(N_3_)]ClO_4_^c^	(**21**)	0.57, 0.65, 0.69	dist. TBP	[44]
[Cu(dpq)_2_(N_3_)]ClO_4_	(**22**)	0.22	dist. SP	[45]
[Cu(pbpd)(N_3_)]PF_6_	(**23**)	0.60	dist. SP	[46]
[Cu(pfbd)(N_3_)]ClO_4_	(**24**)	0.50	Intermed.	[46]
[Cu(Me_3_tren)(N_3_)]ClO_4_	(**25**)	0.80	dist. TBP	[29]
[Cu(isp_3_tren)(N_3_)]ClO_4_	(**3**)	0.78	dist. TBP	This work
[Cu(pdpa)(N_3_)]ClO_4_	(**26**)	0.60	dist. TBP	[29]
[Cu(TPA)(N_3_)]ClO_4_	(**27**)	0.91	dist. TBP	[41]
[Cu(BA)(N_3_)]ClO_4_	(**28**)	0.65	dist. TBP	[47]
[Cu(F_5_BA)(N_3_)]CF_3_SO_3_^c,d^	(**29**)	0.73; 0.68; 0.50; 0.61; 0.31	dist. TBP, dist. SP, Intermed.	[47]
[Cu(pmap)(N_3_)]ClO_4_	(**30**)	0.10	dist. SP	[43]
[Cu(tedmpza)(N_3_)]ClO_4_	(**31**)	0.06	dist. SP	[23]
[Cu(trpn)(N_3_)]ClO_4_	(**32**)	0.23	dist. SP	[48]
[Cu(pmedien)(dca)_2_]	(**33**)	0.23	dist. SP	[29]
[Cu(pzdepy)(dca)]ClO_4_	(**34**)	0.24	dist. SP	[28]
[Cu(tren)(dca)]ClO_4_	(**35**)	0.87	dist. TBP	[27]
[Cu(isp_3_tren)(dca)]ClO_4_	(**4**)	0.77	dist. TBP	This work
[Cu(TPA)(dca)]ClO_4_	(**36**)	0.95	dist. TBP	[28]
[Cu(tepa)(dca)]ClO_4_	(**37**)	0.21	dist. SP	[28]
[Cu(tepza)(dca)]ClO_4_^c^	(**38**)	0.61, 0.63	dist. TBP	[25]
[Cu(tedmpza)(dca)]ClO_4_·0.67H_2_O ^c^	(**6**)	0.79, 0.89, 0.82	dist. TBP	This work

^a^ Ligand abbreviations: L^1^ = [(2-pyridyl)-2-ethyl)-(3,4-dimethoxy)-2-methylpyridyl]methylamine, L^2^ = [(2-pyridyl)-2-ethyl)-(3,5-dimethyl-4-methoxy)-2-methylpyridyl]methylamine, L^3^ = bis(2-ethyl-di(3,5-dimethyl-1*H*-pyrazol-1-yl)-[2-(3,4-dimethoxypyridylmethyl)]amine, L^4^ = bis(2-methyl-pyridyl)-[2-(3,4-dimethoxypyridyl-methyl)]amine, L^5^ = L [*N*,*N*′-bis(1-pyridin-2-yl-ethylidene)-2,2-dimethylpropane-1,3-diamine, bedmpza = bis[2-(di-2,5-dimethyl-1-pyrazolyl)-ethyl)]amine, Mebedmpza = *N*-methyl-bis(3,5-dimethyl-2-(pyrazol-1*H*-yl)ethyl)amine, Bzbedmpza = *N*-benzyl-bis(3,5-dimethyl-2-(pyrazol-1*H*-yl)ethyl)amine, bdmpzpy = 2,6-bis[(2,5-dimethyl-1*H*-pyrazol-1-yl)methyl]pyridine, pmedien = *N*,*N*,*N*′,*N*″,*N*″-pentamethyldiethylenetriamine, DPA = bis(2-methylpyridyl)amine, Me_3_dpt = bis(*N*-methyl-3-aminpropyl)-methylamine, mpipy = *N*-methyl-*N*-(2-pyridylmethyl)piperazine, mpiq = *N*-methyl-*N*-(2-quinolyl-methyl)piperazine, pdpa = [*N*-(3-aminopropyl)-*N*,*N*-bis(2-pyridylmethyl)]amine, pzdepy = *N*,*N*-bis[2-(2-pyridylethyl)]-piperazine, tren = tris(2-aminoethyl)amine, Me_3_tren = tris(2-amino-*N*-methylethyl)amine, isp_3_tren = tris[(2-isopropyl-amino)ethyl)]amine, trpn = tris(3-aminopropyl)amine, TPA = tris[(2-pyridyl)-methyl]-amine, tepza = tris[(2-ethyl-(1*H*-pyrazol-1-yl)]amine, tedmpza = tris(3,5-dimethyl-ethyl-1*H*-pyrazol-1-yl)amine, pzdepy = 1,4-bis[2-(2-pyridyl-ethyl)]piperazine, bdmpe = *N*,*N*-bis((3,5-dimethyl-1*H*-pyrazol-yl)methyl)-2-(phenylthio)ethan-1-amine, cyclen-tpam = 1,4,7,10-tetrakis-(propionamide)-1,4,7,10-tetraazacyclododecane, dpq = dipyrido[3,2-f: 20,30-h]-quinoxaline, pbpd = *N*,*N*′-(bis(pyridin-2-yl)benzylidene)-propane-1,3-diamine, pfbd = *N*,*N*′-(bis(pyridin-2-yl)-formylidene)butane-1,4-diamine, BA = 6-((bis(pyridin-2-ylmethyl)amino)methyl)-*N*-((phenyl)-methyl)pyridin-2-amine, F_5_BA = 6-((bis(pyridin-2-ylmethyl)amino)methyl)-*N*-((perfluorophenyl)-methyl)pyridin-2-amine. ^b^ dist. = distorted. Intermed. = intermediate. ^c^ two or more molecules are in the unit cell. ^d^ complex is severely disorderd. *Note*: As in the title compounds **1**–**6** the CuN5 complexes all 10 valence angles of N-Cu-N have different values, the maximum angle is always the θ angle, and next largest angle is always the ϕ angle.

**Table 2:** The Visible spectral data in mononuclear five-coordinated pseudohalido-copper(II) complexes in solutions and the assigned predominant stereochemical geometry.

Complex		Solvent	λ_max_, nm (ε_max_, M^−1^cm^−1^)	Predicted Geometry	Ref.
[Cu(L^1^)(NCS)_2_]	(**1**)	CH_3_CN	676 (190)	dist. SP	This work
[Cu(L^2^)(NCS)_2_]	(**2**)	CH_3_CN	676 (196)	dist. SP	This work
[Cu(bdmpzpy)(NCS)_2_]	(**8**)	CH_3_OH	670 (sat)	dist. SP	[23]
[Cu(Mebedmpza)(NCS)_2_]	(**9**)	CH_3_CN	731 (155, br)	dist. SP	[22]
[Cu(Bzbedmpza)(NCS)_2_]	(**10**)	CH_3_CN	704 (155, br)	dist. SP	[22]
[Cu(mpipy)(NCS)_2_]	(**12**)	CH_3_CN	729 (288)	dist. SP	[42]
[Cu(mpiq)(NCS)_2_]	(**13**)	CH_3_CN	775 (230)	dist. SP	[42]
[Cu(pzdepy)(NCS)]ClO_4_	(**14**)	H_2_O	591 (304)	dist. SP	[43]
[Cu(Me_3_dpt)(NCS)_2_]	(**15**)	CH_3_CN	686 (204), 932 (141)	dist. SP	[40]
[Cu(bedmpza)(NCS)_2_]	(**16**)	CH_3_CN	725 (149)	dist. SP	[24]
[Cu(tepza)(NCS)]ClO_4_	(**17**)	CH_3_CN	704 (174, br)	dist. SP	[25]
[Cu(bdmpe)(NCS)]ClO_4_	(**18**)	CH_3_CN	663 (174)	dist. SP	[30]
[Cu(L^3^)(NCS)]ClO_4_	(**5**)	CH_3_CN	~630 (sh), ~940 (76)	dist. SP	This work
[Cu(cyclen-tpam)(NCS)]ClO_4_·3H_2_O	(**19**)	H_2_O	623 (435)	dist. SP	[23]
[Cu(L^5^)(NCS)]ClO_4_	(**20**)	CH_3_CN	720 (203)	dist. SP	[44]
[Cu(L^5^)(N_3_)]ClO_4_	(**21**)	CH_3_CN	383 (155)	dist. SP	[44]
[Cu(dpq)_2_(N_3_)]ClO_4_	(**22**)	DMSO	680 (110), 1050 (sh)	dist. SP	[45]
[Cu(pbpd)(N_3_)]PF_6_	(**23**)	MeOH	718	dist. SP	[46]
[Cu(pfbd)(N_3_)]ClO_4_	(**24**)	MeOH	720	dist. SP	[46]
[Cu(Me_3_tren)(N_3_)]ClO_4_	(**25**)	H_2_O	665 (~162), 855 (300, br)	dist. TBP	[29]
[Cu(isp_3_tren)(N_3_)]ClO_4_	(**3**)	CH_3_CN	695 (364, br), 870 (397, br)	dist. TBP	This work
[Cu(pdpa)(N_3_)]ClO_4_	(**26**)	H_2_O	656 (132, sh), 852 (200)	dist. TBP	[29]
[Cu(TPA)(N_3_)]ClO_4_	(**27**)	CH_3_OH	~650, 836 (201)	dist. TBP	[41]
[Cu(BA)(N_3_)]ClO_4_	(**28**)	CH_3_CN	650, ~880	~dist. TBP	[47]
[Cu(F_5_BA)(N_3_)]ClO_4_	(**29**)	CH_3_CN	655, ~870	~dist. TBP	[47]
[Cu(pmap)(N_3_)]ClO_4_	(**30**)	H_2_O	634 (268)	dist. SP	[43]
[Cu(tedmpza)(N_3_)]ClO_4_	(**31**)	CH_3_CN	713 (354, br)	dist. SP	[23]
[Cu(trpn)(N_3_)]ClO_4_	(**32**)	H_2_O	685 (118), 900 (sh)	dist. SP	[48]
[Cu(trpn)(N_3_)]ClO_4_	(**32**)	DMSO	670 (275), 920 (sh)	dist. SP	[48]
[Cu(pmedien)(dca)_2_]	(**33**)	H_2_O	638 (265)	dist. SP	[29]
[Cu(pzdepy)(dca)]ClO_4_	(**34**)	H_2_O	591 (304)	dist. SP	[28]
[Cu(tren)(dca)]ClO_4_	(**35**)	CH_3_CN	665, 828	dist. TBP	[27]
[Cu(isp_3_tren)(dca)]ClO_4_	(**4**)	CH_3_CN	~660 (sh), 837 (397, br)	dist. TBP	This work
[Cu(TPA)(dca)]ClO_4_	(**36**)	H_2_O	~650, 872 (239)	dist. TBP	[28]
[Cu(tepa)(dca)]ClO_4_	(**37**)	H_2_O	650 (114), ~739 (sh)	dist. SP	[28]
[Cu(tepa)(dca)]ClO_4_	(**37**)	CH_3_OH	640 (187)	dist. SP	[28]
[Cu(tepa)(dca)]ClO_4_	(**37**)	CH_3_CN	645 (217)	dist. SP	[28]
[Cu(tepa)(dca)]ClO_4_	(**37**)	CH_3_NO_2_	640 (229)	dist. SP	[28]
[Cu(tepza)(dca)]ClO_4_	(**38**)	H_2_O	676 (93, br)	dist. SP	[25]
[Cu(tedmpza)(dca)]ClO_4_·0.67H_2_O	(**6**)	CH_3_CN	~730 (90, sh), ~940 (110, br)	dist. TBP	This work
[Cu(L^4^)(dca)](ClO_4_)·2H_2_O	(**7**)	CH_3_CN	~665 (sh), 880 (256, br)	dist. TBP	This work

**Table 3:** The molecular structure, the distortion index parameter (τ), the visible spectral absorption bands of mononuclear five-coordinated halido-Cu(II) complexes ^a^ in acetonitrile solutions and the assigned geometry.

Complex		τ Value	Geometry	λ_max_, nm (ε_max_, M^−1^cm^−1^)	Assigned Geometry	Ref.
[Cu(TPA)Cl]ClO_4_·½H_2_O ^b,c^	(**39**)	0.98, 0.94	TBP	~730 (sh), 950 (br)	dist TBP	[52]
[Cu(TPA)Cl]PF_6_	(**40**)	≈1.0	TBP	962 (210), 632 (88, sh)	TBP	[53,54]
[Cu(TPA)F]PF_6_·CH_2_Cl_2_	(**41**)	0.91	dist. TBP	710 (sh), 872	dist. TBP	[54]
[Cu(6-MeTPA)Cl]PF_6_	(**42**)	0.12	dist. SP	~670 (sh), 885 (132, br)	dist TBP	[8]
[Cu(6-MeTPA)Cl]ClO_4_^c^	(**43**)	0.16, 0.24	dist. SP	-		[55]
[Cu(6-Me_2_TPA)Cl]PF_6_	(**44**)	-	-	685 (148), ~860 (sh)	dist TBP	[8]
[Cu(6-Me_2_TPA)Cl]ClO_4_	(**45**)	0.07	SP	-	-	[55]
[Cu(6-Me_3_TPA)Cl]ClO_4_	(**46**)	0.43	Intermed.	-	-	[55]
[Cu(pmea)Cl]ClO_4_·H_2_O	(**47**)	≈0	SP	-		[56]
[Cu(pmapCl]ClO_4_	(**48**)	≈0	SP	-		[56,57]
[Cu(pmap)Cl]PF_6_·¼CH_2_Cl_2_	(**49**)	-	dist. SP	639 (192), 898 (sh, 23)	dist SP	[56]
[Cu(tepa)Cl]PF_6_	(**50**)	≈0	SP	665 (200), 967 (48)	SP	[57]
[Cu(BPQA)Cl]ClO_4_	(**51**)	0.16	dist. SP	~640, 880 (151, br)	dist TBP	[8]
[Cu(BPQA)Cl]PF_6_	(**52**)	0.13	dist. SP	~700 (sh), 900 (159, br)	dist TBP	[8]
[Cu(BQPA)Cl]ClO_4_	(**53**)	-	-	730 (138, br), ~880 (143, br)	Intermed.	[8]
[Cu(BQPA)Cl]PF_6_	(**54**)	0.64	dist. TBP	~660 (sh), 880 (141, br)	dist TBP	[8]
[Cu(L^6^)Cl]ClO_4_	(**55**)	-	-	~725 (sh), 955 (221)	dist TBP	[8]
[Cu(L^6^)Cl]PF_6_^c^	(**56**)	0.96 (mean)	TBP	~700, ~850 (sh), 970 (355)	dist TBP	[8]
[Cu(L^7^)Cl]ClO_4_	(**57**)	0.86	dist. TBP	~715 (sh), 960 (230, br)	dist TBP	[8]
[Cu(L^8^)Cl]ClO_4_	(**58**)	0.80	dist. TBP	~710, ~880 (sh), 970 (371)	dist TBP	[8]
[Cu(Me_6_tren)Cl]Cl	(**59**)	1.0	TBP	938 (451)	dist TBP	[58]
[Cu(Me_6_tren)Br]Br	(**60**)	1.0	TBP	973 (426)	dist TBP	[58]
[Cu(L^9^)Cl_2_] ^d^	(**61**)	0.11	dist. SP	787 (280)	dist. SP	[49]
[Cu(L^10^)(H_2_O)(Cl)_2_] ^e^	(**62**)	0.27	dist. SP	463 (sh, 503), 806 (135)	dist. SP	[50]
[Cu(L^11^)(Br)_2_]	(**63**)	0.09	dist. SP	-		[51]

^a^ TPA = tris[(2-pyridyl)methyl]amine, pmea = [bis((2-pyridyl)methyl)-2-(2-pyridyl)-ethyl]amine, pmpa = [bis((2-(2-pyridyl)ethyl))-(2-pyridyl)-methyl]amine, tepa = tris[2-(2-pyridyl)ethyl]amine, 6-MeTPA = ((6-methyl-2-pyridyl)methyl)bis(2-pyridylmethyl)amine, 6-Me_2_TPA = bis(6-methyl-2-pyridyl)methyl)-(2-pyridylmethyl)amine, BPQA = bis(2-pyridylmethyl)-(2-quinolylmethyl)amine and BQPA = bis(2-quinolylmethyl)-(2-pyridylmethyl)amine, L^6^ = [(3,5-dimethyl-4-methoxy-2-pyridylmethyl)-bis(2-pyridyl-methyl)]amine, L^7^ = [(3,4-dimethoxy-2-pyridylmethyl)-bis(2-pyridylmethyl)]amine, L^8^ =[bis(3,5-dimethyl-4-methoxy-2-pyridylmethyl)-(2-pyridylmethyl)]amine, Me_6_tren = tris[2-(dimethylamino)ethyl]-amine, L^9^ = benzyldipicolylamine; L^10^ = 2,9-di-methylphenanthroline; L^11^ = 2-(6-(pyridine-2-yl)-4-*p*-tolylpyridin-2-yl)pyridine. ^b^ measured in aqueous solution. ^c^ there are two more molecules in the unit cell. ^d^ measured in DMF. ^e^ measured in DMSO.

**Table 4:** Crystallographic data and processing parameters of **1**–**6**.

Compound	1	2	3
Empirical formula	C_18_H_21_CuN_5_O_2_S_2_	C_19_H_23_CuN_5_OS_2_	C_15_H_36_ClCuN_7_O_4_
Formula mass	467.01	465.09	477.51
System	Monoclinic	Monoclinic	Orthorhombic
Space group	P2_1_/c	P2_1_/c	P2_1_2_1_2_1_
a (Å)	9.9325(18)	8.037(3)	8.2797(3)
b (Å)	7.6958(14)	28.198(9)	10.3404(4)
c (Å)	26.491(5)	9.219(3)	25.1838(10)
α (°)	90	90	90
β (°)	95.923(3)	93.416(5)	90
γ (°)	90	90	90
V (Å^3^)	2014.8(6)	2085.6(12)	2156.12(14)
Z	4	4	4
T (K)	100(2)	100(2)	100(2)
μ (mm^−^^1^)	1.316	1.267	1.173
D_calc_ (Mg/m^3^)	1.540	1.481	1.471
θ max (°)	26.349	26.395	24.999
Data collected	14,125	12,796	71,092
Unique refl./R_int_	4099/0.0268	4193/0.1191	3756/0.0437
Parameters/Restraints	256/0	257/0	260/0
Goodness-of-Fit on F^2^	1.074	1.123	1.110
R1/wR2 (all data)	0.0373/0.0956	0.0943/0.2051	0.0152/0.0388
Residual extrema (e/Å^3^)	1.119/−0.465	1.215/−0.998	0.210/−0.150
**Compound**	**4**	**5**	**6**
Empirical formula	C_17_H_36_ClCuN_7_O_4_	C_23_H_32_ClCuN_7_O_6_S	C_69_H_103_Cl_3_Cu_3_N_30_O_14_
Formula mass	501.53	633.62	1873.81
System	Orthorhombic	Monoclinic	Triclinic
Space group	Pbca	P2_1_/c	P1
a (Å)	20.0498(9)	8.8023(7)	9.9990(10)
b (Å)	15.1408(6)	29.700(2)	15.7623(17)
c (Å)	15.5606(7)	11.2141(10)	15.9456(18)
α (°)	90	90	114.689(5)
β (°)	90	93.260(4)	100.284(5)
γ (°)	90	90	103.717(5)
V (Å^3^)	4723.7(4)	2926.9(4)	2105.8(4)
Z	8	4	1
T (K)	100(2)	100(2)	100(2)
μ (mm^−^^1^)	1.074	0.958	0.925
D_calc_ (Mg/m^3^)	1.410	1.438	1.478
θ max (°)	24.997	27.025	28.276
Data collected	102,653	25,071	93,026
Unique refl./R_int_	4125/0.1406	6395/0.0647	17,329/0.0922
Parameters/Restraints	415/0	395/36	1099/12
Goodness-of-Fit on F^2^	1.106	1.062	1.044
R1/wR2 (all data)	0.0422/0.1352	0.0542/0.1258	0.0549/0.1347
Residual extrema (e/Å^3^)	1.180/−0.477	0.679/−0.526	1.249/−0.942

## Supplementary Materials

The following are available online. CDCC 2008941-2008946 contains the supplementary crystallographic data for **1**–**6**, respectively. These data can be obtained free of charge via http//www.ccdc.cam.ac.uk/conts/retrieving.html, or from the Cambridge Crystallographic Data Centre, 12 Union Road, Cambridge CB2 1EZ, UK; fax: (+44) 1223-336-033; or e-mail: deposit@ccdc cam.ac.uk. Selected bond lengths (Å) and angles (°) for the compounds under investigation are given in Tables S1–S3 and their corresponding packing views are shown in Figures S1–S6 for **1**–**6** coordination compounds, respectively. Coordination figures of Cu1 and Cu3 of **6** are given in Figures S7 and S8, respectively. The UV-vis spectra of the seven complexes under investigation are given in Figures S9–S15. Possible hydrogen bonds for compounds **1**–**6** are shown in Table S4.
