# Supplementary material for: Five-Coordinated Geometries from Molecular Structures to Solutions in Copper(II) Complexes Generated from Polydentate-N-Donor Ligands and Pseudohalides
Source: Molecules. 2020 Jul 25;25(15):3376. doi: 10.3390/molecules25153376 (PMC7436159; doi:10.3390/molecules25153376)

checkCIF/PLATON report

Structure factors have been supplied for datablock(s) s2955a

THIS REPORT IS FOR GUIDANCE ONLY. IF USED AS PART OF A REVIEW PROCEDURE FOR PUBLICATION, IT SHOULD NOT REPLACE THE EXPERTISE OF AN EXPERIENCED CRYSTALLOGRAPHIC REFEREE.

No syntax errors found. CIF dictionary Interpreting this report

Datablock: s2955a

```
Bond precision:  C-C = 0.0036 A          Wavelength=0.71073

Cell:            a=9.9325(18)     b=7.6985(14)       c=26.491(5)
                 alpha=90         beta=95.923(3)     gamma=90
Temperature:     100 K

                Calculated                 Reported
Volume          2014.8(6)                  2014.9(6)
Space group     P 21/c                     P 21/c
Hall group      -P 2ybc                    -P 2ybc
Moiety formula  C18 H21 Cu N5 O2 S2        C18 H21 Cu N5 O2 S2
Sum formula     C18 H21 Cu N5 O2 S2        C18 H21 Cu N5 O2 S2
Mr              467.07                     467.06
Dx,g cm-3       1.540                      1.540
Z               4                          4
Mu (mm-1)       1.316                      1.316
F000            964.0                      964.0
F000'           966.51
h,k,lmax        12,9,33                    12,9,33
Nref            4122                       4099
Tmin,Tmax       0.646,0.877                0.623,0.944
Tmin'           0.633

Correction method= # Reported T Limits: Tmin=0.623 Tmax=0.944
AbsCorr = MULTI-SCAN

Data completeness= 0.994          Theta(max)= 26.349

R(reflections)= 0.0373( 3820)     wR2(reflections)= 0.0956( 4099)

S = 1.074                  Npar= 256
```

The following ALERTS were generated. Each ALERT has the format
test-name_ALERT_alert-type_alert-level.
Click on the hyperlinks for more details of the test.

Alert level C

```
PLAT094_ALERT_2_C Ratio of Maximum / Minimum Residual Density ....       2.41 Report
PLAT906_ALERT_3_C Large K Value in the Analysis of Variance ......      2.396 Check
PLAT911_ALERT_3_C Missing FCF Refl Between Thmin & STh/L=    0.600         13 Report
```

Alert level G

```
PLAT230_ALERT_2_G Hirshfeld Test Diff for    S2       --C2       .        6.0 s.u.
PLAT793_ALERT_4_G Model has Chirality at N4          (Centro SPGR)          R Verify
PLAT794_ALERT_5_G Tentative Bond Valency for Cu1       (I)       .       1.21 Info
PLAT883_ALERT_1_G No Info/Value for _atom_sites_solution_primary .     Please Do !
PLAT912_ALERT_4_G Missing # of FCF Reflections Above STh/L=  0.600         11 Note
PLAT941_ALERT_3_G Average HKL Measurement Multiplicity ..........         3.4 Low
PLAT955_ALERT_1_G Reported (CIF) and Actual (FCF) Lmax Differ by .          1 Units
PLAT978_ALERT_2_G Number C-C Bonds with Positive Residual Density.          0 Info
PLAT992_ALERT_5_G Repd & Actual _reflns_number_gt Values Differ by          1 Check
```

```
   0 ALERT level A = Most likely a serious problem - resolve or explain
   0 ALERT level B = A potentially serious problem, consider carefully
   3 ALERT level C = Check. Ensure it is not caused by an omission or oversight
   9 ALERT level G = General information/check it is not something unexpected

   2 ALERT type 1 CIF construction/syntax error, inconsistent or missing data
   3 ALERT type 2 Indicator that the structure model may be wrong or deficient
   3 ALERT type 3 Indicator that the structure quality may be low
   2 ALERT type 4 Improvement, methodology, query or suggestion
   2 ALERT type 5 Informative message, check
```

It is advisable to attempt to resolve as many as possible of the alerts in all categories. Often the minor alerts point to easily fixed oversights, errors and omissions in your CIF or refinement strategy, so attention to these fine details can be worthwhile. In order to resolve some of the more serious problems it may be necessary to carry out additional measurements or structure refinements. However, the purpose of your study may justify the reported deviations and the more serious of these should normally be commented upon in the discussion or experimental section of a paper or in the "special_details" fields of the CIF. checkCIF was carefully designed to identify outliers and unusual parameters, but every test has its limitations and alerts that are not important in a particular case may appear. Conversely, the absence of alerts does not guarantee there are no aspects of the results needing attention. It is up to the individual to critically assess their own results and, if necessary, seek expert advice.

Publication of your CIF in IUCr journals

A basic structural check has been run on your CIF. These basic checks will be run on all CIFs submitted for publication in IUCr journals (*Acta Crystallographica*, *Journal of Applied Crystallography*, *Journal of Synchrotron Radiation*); however, if you intend to submit to *Acta Crystallographica Section C* or *E* or *IUCrData*, you should make sure that full publication checks are run on the final version of your CIF prior to submission.

Publication of your CIF in other journals

Please refer to the *Notes for Authors* of the relevant journal for any special instructions relating to CIF submission.

PLATON version of 22/04/2020; check.def file version of 09/03/2020

Datablock s2955a - ellipsoid plot

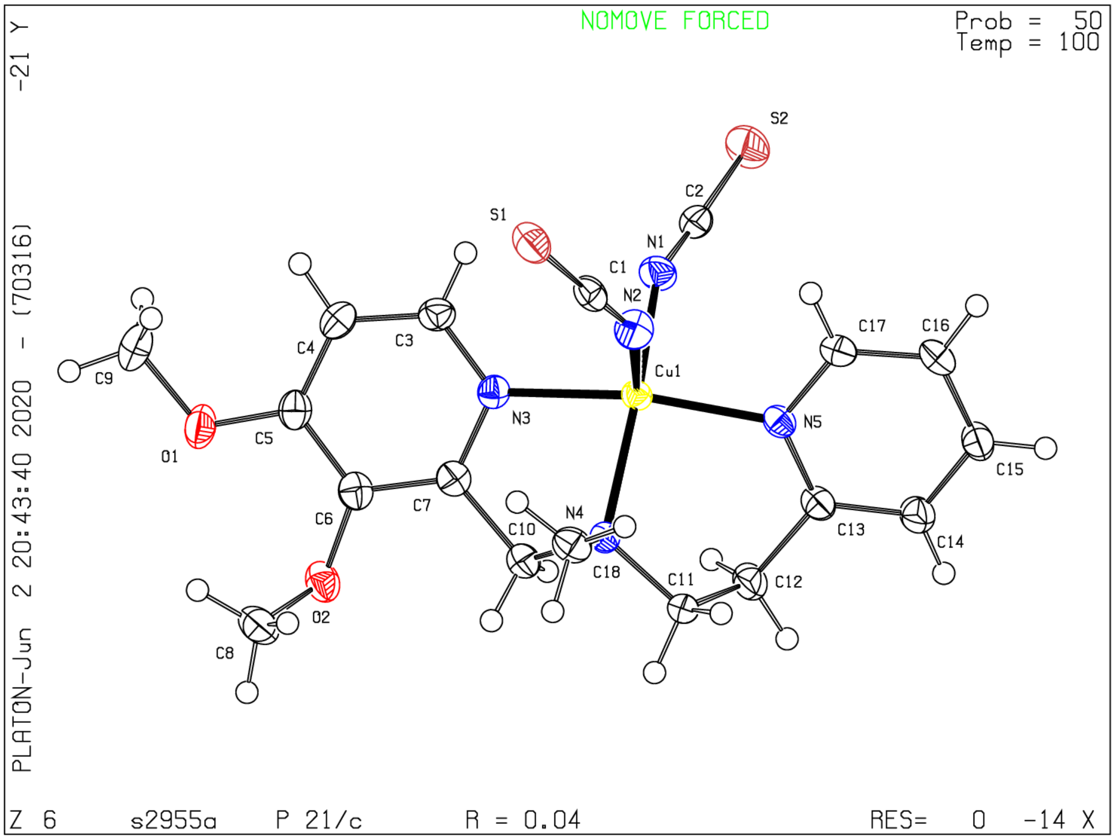

checkCIF/PLATON report

Structure factors have been supplied for datablock(s) s2949a

THIS REPORT IS FOR GUIDANCE ONLY. IF USED AS PART OF A REVIEW PROCEDURE FOR PUBLICATION, IT SHOULD NOT REPLACE THE EXPERTISE OF AN EXPERIENCED CRYSTALLOGRAPHIC REFEREE.

No syntax errors found. CIF dictionary Interpreting this report

Datablock: s2949a

```
Bond precision:  C-C = 0.0102 A          Wavelength=0.71073

Cell:             a=8.037(3)     b=28.198(9)         c=9.219(3)
                  alpha=90       beta=93.416(5)      gamma=90
Temperature:      100 K

                Calculated                Reported
Volume          2085.6(12)                2085.6(12)
Space group     P 21/c                    P 21/c
Hall group      -P 2ybc                   -P 2ybc
Moiety formula  C19 H23 Cu N5 O S2        C19 H23 Cu N5 O S2
Sum formula     C19 H23 Cu N5 O S2        C19 H23 Cu N5 O S2
Mr              465.09                    465.08
Dx,g cm-3       1.481                     1.481
Z               4                         4
Mu (mm-1)       1.267                     1.267
F000            964.0                     964.0
F000'           966.48
h,k,lmax        10,35,11                  10,35,11
Nref            4269                      4193
Tmin,Tmax       0.808,0.904               0.623,0.965
Tmin'           0.618

Correction method= # Reported T Limits: Tmin=0.623 Tmax=0.965
AbsCorr = MULTI-SCAN

Data completeness= 0.982          Theta(max)= 26.395

R(reflections)= 0.0943( 2992)     wR2(reflections)= 0.2051( 4193)

S = 1.123                   Npar= 257
```

The following ALERTS were generated. Each ALERT has the format
test-name_ALERT_alert-type_alert-level.
Click on the hyperlinks for more details of the test.

Alert level C

```
PLAT341_ALERT_3_C Low Bond Precision on  C-C Bonds ...............    0.01015 Ang.
PLAT906_ALERT_3_C Large K Value in the Analysis of Variance ......     20.079 Check
PLAT906_ALERT_3_C Large K Value in the Analysis of Variance ......      4.010 Check
PLAT911_ALERT_3_C Missing FCF Refl Between Thmin & STh/L=    0.600         33 Report
PLAT977_ALERT_2_C Check Negative Difference Density on H10B             -0.48 eA-3
```

Alert level G

```
PLAT083_ALERT_2_G SHELXL Second Parameter in WGHT  Unusually Large       6.19 Why ?
PLAT793_ALERT_4_G Model has Chirality at N4          (Centro SPGR)          R Verify
PLAT794_ALERT_5_G Tentative Bond Valency for Cu2       (I)       .       1.22 Info
PLAT883_ALERT_1_G No Info/Value for _atom_sites_solution_primary .     Please Do !
PLAT910_ALERT_3_G Missing # of FCF Reflection(s) Below Theta(Min).          1 Note
PLAT912_ALERT_4_G Missing # of FCF Reflections Above STh/L=  0.600         42 Note
PLAT933_ALERT_2_G Number of OMIT Records in Embedded .res File ...          1 Note
PLAT941_ALERT_3_G Average HKL Measurement Multiplicity ...........        3.1 Low
PLAT978_ALERT_2_G Number C-C Bonds with Positive Residual Density.          1 Info
```

0 **ALERT level A** = Most likely a serious problem - resolve or explain
0 **ALERT level B** = A potentially serious problem, consider carefully
5 **ALERT level C** = Check. Ensure it is not caused by an omission or oversight
9 **ALERT level G** = General information/check it is not something unexpected

1 ALERT type 1 CIF construction/syntax error, inconsistent or missing data
4 ALERT type 2 Indicator that the structure model may be wrong or deficient
6 ALERT type 3 Indicator that the structure quality may be low
2 ALERT type 4 Improvement, methodology, query or suggestion
1 ALERT type 5 Informative message, check

It is advisable to attempt to resolve as many as possible of the alerts in all categories. Often the minor alerts point to easily fixed oversights, errors and omissions in your CIF or refinement strategy, so attention to these fine details can be worthwhile. In order to resolve some of the more serious problems it may be necessary to carry out additional measurements or structure refinements. However, the purpose of your study may justify the reported deviations and the more serious of these should normally be commented upon in the discussion or experimental section of a paper or in the "special_details" fields of the CIF. checkCIF was carefully designed to identify outliers and unusual parameters, but every test has its limitations and alerts that are not important in a particular case may appear. Conversely, the absence of alerts does not guarantee there are no aspects of the results needing attention. It is up to the individual to critically assess their own results and, if necessary, seek expert advice.

Publication of your CIF in IUCr journals

A basic structural check has been run on your CIF. These basic checks will be run on all CIFs submitted for publication in IUCr journals (*Acta Crystallographica*, *Journal of Applied Crystallography*, *Journal of Synchrotron Radiation*); however, if you intend to submit to *Acta Crystallographica Section C* or *E* or *IUCrData*, you should make sure that full publication checks are run on the final version of your CIF prior to submission.

Publication of your CIF in other journals

Please refer to the *Notes for Authors* of the relevant journal for any special instructions relating to CIF submission.

PLATON version of 22/04/2020; check.def file version of 09/03/2020

Datablock s2949a - ellipsoid plot

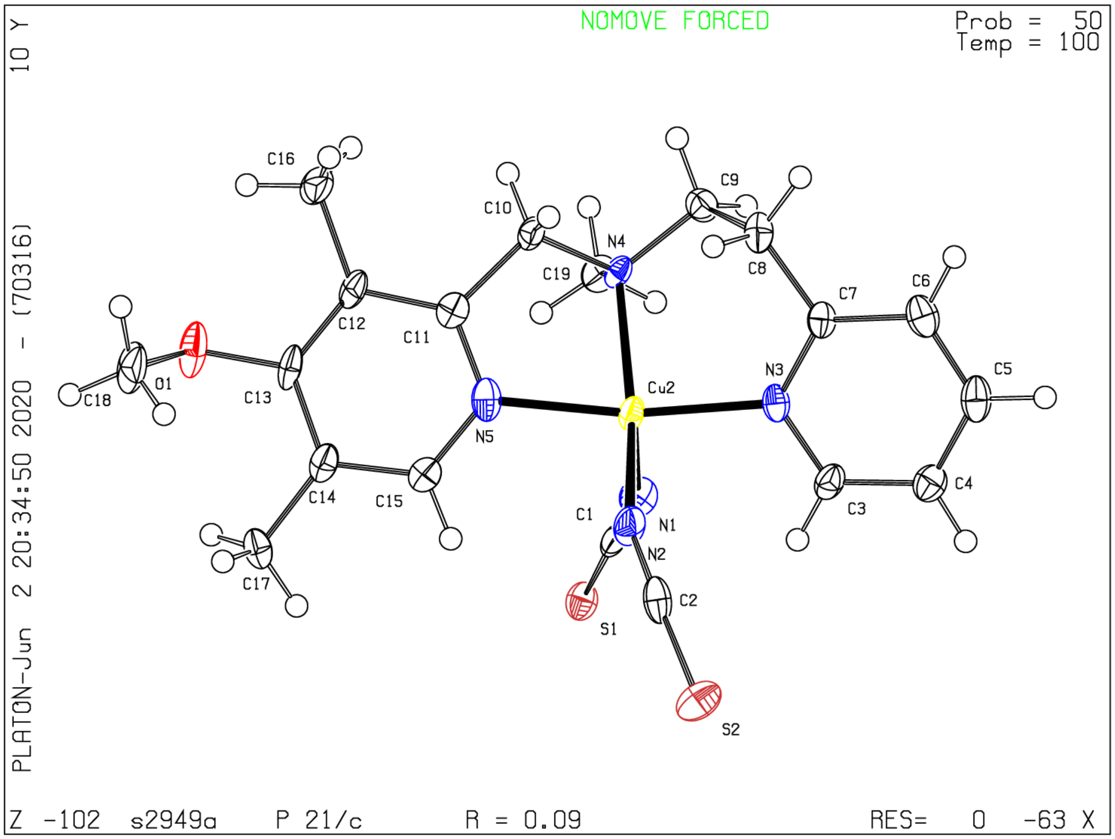

checkCIF/PLATON report

Structure factors have been supplied for datablock(s) AT801_a

THIS REPORT IS FOR GUIDANCE ONLY. IF USED AS PART OF A REVIEW PROCEDURE FOR PUBLICATION, IT SHOULD NOT REPLACE THE EXPERTISE OF AN EXPERIENCED CRYSTALLOGRAPHIC REFEREE.

No syntax errors found. CIF dictionary Interpreting this report

Datablock: AT801_a

```
Bond precision:  C-C = 0.0030 A          Wavelength=0.71073

Cell:            a=8.2797(3)     b=10.3404(4)     c=25.1838(10)
                 alpha=90        beta=90          gamma=90
Temperature:     100 K

                Calculated                  Reported
Volume          2156.12(14)                 2156.12(14)
Space group     P 21 21 21                  P 21 21 21
Hall group      P 2ac 2ab                   P 2ac 2ab
Moiety formula  C15 H36 Cu N7, Cl O4        C15 H36 Cu N7, Cl O4
Sum formula     C15 H36 Cl Cu N7 O4         C15 H36 Cl Cu N7 O4
Mr              477.51                      477.50
Dx,g cm-3       1.471                       1.471
Z               4                           4
Mu (mm-1)       1.173                       1.173
F000            1012.0                      1012.0
F000'           1014.16
h,k,lmax        9,12,29                     9,12,29
Nref            3789[ 2189]                 3756
Tmin,Tmax       0.945,0.954                 0.610,0.746
Tmin'           0.943

Correction method= # Reported T Limits: Tmin=0.610 Tmax=0.746
AbsCorr = MULTI-SCAN

Data completeness= 1.72/0.99      Theta(max)= 24.999

R(reflections)= 0.0152( 3741)     wR2(reflections)= 0.0388( 3756)

S = 1.110                  Npar= 260
```

The following ALERTS were generated. Each ALERT has the format
test-name_ALERT_alert-type_alert-level.
Click on the hyperlinks for more details of the test.

Alert level C

```
PLAT230_ALERT_2_C Hirshfeld Test Diff for   N5        --N6         .         5.9 s.u.
PLAT480_ALERT_4_C Long H...A H-Bond Reported H1A       ..CL1               2.92 Ang.
PLAT480_ALERT_4_C Long H...A H-Bond Reported H1A       ..O2                2.63 Ang.
PLAT480_ALERT_4_C Long H...A H-Bond Reported H4C       ..O3                2.61 Ang.
PLAT480_ALERT_4_C Long H...A H-Bond Reported H9B       ..O4                2.64 Ang.
PLAT480_ALERT_4_C Long H...A H-Bond Reported H15C      ..O3                2.61 Ang.
PLAT911_ALERT_3_C Missing FCF Refl Between Thmin & STh/L=    0.595           26 Report
```

Alert level G

```
PLAT007_ALERT_5_G Number of Unrefined Donor-H Atoms ..............           3 Report
PLAT791_ALERT_4_G Model has Chirality at N2          (Chiral SPGR)           R Verify
PLAT791_ALERT_4_G Model has Chirality at N3          (Chiral SPGR)           R Verify
PLAT791_ALERT_4_G Model has Chirality at N4          (Chiral SPGR)           R Verify
PLAT794_ALERT_5_G Tentative Bond Valency for Cu1       (II)      .        2.02 Info
PLAT909_ALERT_3_G Percentage of I>2sig(I) Data at Theta(Max) Still         99% Note
PLAT910_ALERT_3_G Missing # of FCF Reflection(s) Below Theta(Min).           2 Note
PLAT933_ALERT_2_G Number of OMIT Records in Embedded .res File ...          34 Note
PLAT978_ALERT_2_G Number C-C Bonds with Positive Residual Density.           3 Info
```

---

0 **ALERT level A** = Most likely a serious problem - resolve or explain
0 **ALERT level B** = A potentially serious problem, consider carefully
7 **ALERT level C** = Check. Ensure it is not caused by an omission or oversight
9 **ALERT level G** = General information/check it is not something unexpected

0 ALERT type 1 CIF construction/syntax error, inconsistent or missing data
3 ALERT type 2 Indicator that the structure model may be wrong or deficient
3 ALERT type 3 Indicator that the structure quality may be low
8 ALERT type 4 Improvement, methodology, query or suggestion
2 ALERT type 5 Informative message, check

It is advisable to attempt to resolve as many as possible of the alerts in all categories. Often the minor alerts point to easily fixed oversights, errors and omissions in your CIF or refinement strategy, so attention to these fine details can be worthwhile. In order to resolve some of the more serious problems it may be necessary to carry out additional measurements or structure refinements. However, the purpose of your study may justify the reported deviations and the more serious of these should normally be commented upon in the discussion or experimental section of a paper or in the "special_details" fields of the CIF. checkCIF was carefully designed to identify outliers and unusual parameters, but every test has its limitations and alerts that are not important in a particular case may appear. Conversely, the absence of alerts does not guarantee there are no aspects of the results needing attention. It is up to the individual to critically assess their own results and, if necessary, seek expert advice.

Publication of your CIF in IUCr journals

A basic structural check has been run on your CIF. These basic checks will be run on all CIFs submitted for publication in IUCr journals (*Acta Crystallographica*, *Journal of Applied Crystallography*, *Journal of Synchrotron Radiation*); however, if you intend to submit to *Acta Crystallographica Section C* or *E* or *IUCrData*, you should make sure that full publication checks are run on the final version of your CIF prior to submission.

Publication of your CIF in other journals

Please refer to the *Notes for Authors* of the relevant journal for any special instructions relating to CIF submission.

PLATON version of 30/01/2018; check.def file version of 30/01/2018

Datablock AT801_a - ellipsoid plot

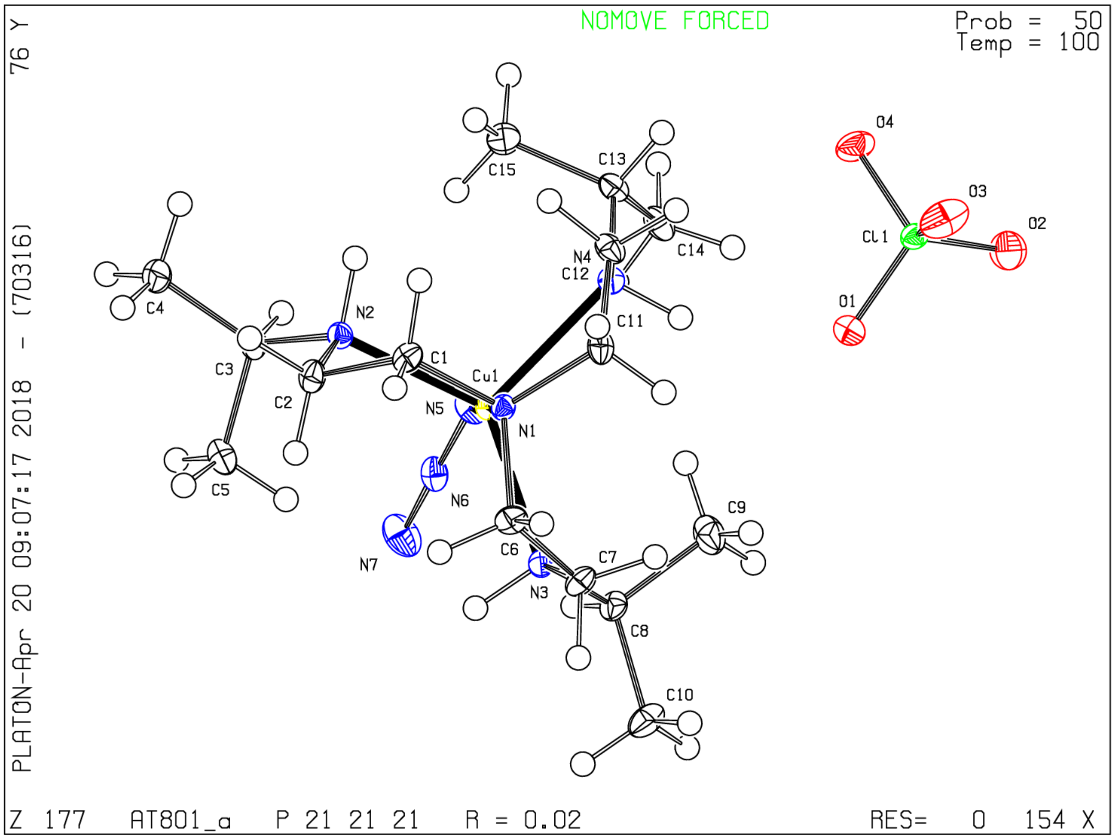

checkCIF/PLATON report

Structure factors have been supplied for datablock(s) AT802_a

THIS REPORT IS FOR GUIDANCE ONLY. IF USED AS PART OF A REVIEW PROCEDURE FOR PUBLICATION, IT SHOULD NOT REPLACE THE EXPERTISE OF AN EXPERIENCED CRYSTALLOGRAPHIC REFEREE.

No syntax errors found. CIF dictionary Interpreting this report

Datablock: AT802_a

```
Bond precision:  C-C = 0.0062 A          Wavelength=0.71073

Cell:            a=20.0498(9)     b=15.1408(6)     c=15.5606(7)
                 alpha=90         beta=90          gamma=90
Temperature:     100 K

                Calculated                  Reported
Volume          4723.7(4)                   4723.7(4)
Space group     P b c a                     P b c a
Hall group      -P 2ac 2ab                  -P 2ac 2ab
Moiety formula  C17 H36 Cu N7, Cl O4        ?
Sum formula     C17 H36 Cl Cu N7 O4         C17 H36 Cl Cu N7 O4
Mr              501.53                      501.52
Dx,g cm-3       1.410                       1.410
Z               8                           8
Mu (mm-1)       1.074                       1.074
F000            2120.0                      2120.0
F000'           2124.36
h,k,lmax        23,18,18                    23,18,18
Nref            4146                        4125
Tmin,Tmax       0.968,0.968                 0.528,0.741
Tmin'           0.968

Correction method= # Reported T Limits: Tmin=0.528 Tmax=0.741
AbsCorr = MULTI-SCAN

Data completeness= 0.995          Theta(max)= 24.997

R(reflections)= 0.0422( 3091)     wR2(reflections)= 0.1352( 4125)

S = 1.106                  Npar= 415
```

The following ALERTS were generated. Each ALERT has the format
test-name_ALERT_alert-type_alert-level.
Click on the hyperlinks for more details of the test.

Alert level C

```
RINTA01_ALERT_3_C  The value of Rint is greater than 0.12
            Rint given   0.141
PLAT088_ALERT_3_C Poor Data / Parameter Ratio ....................       9.94 Note
PLAT094_ALERT_2_C Ratio of Maximum / Minimum Residual Density ....       2.47 Report
PLAT222_ALERT_3_C Non-Solv.  Resd 1  H   Uiso(max)/Uiso(min) Range       10.0 Ratio
PLAT244_ALERT_4_C Low    'Solvent' Ueq as Compared to Neighbors of        Cl1 Check
PLAT245_ALERT_2_C U(iso) H12       Smaller than U(eq) C12       by      0.012 Ang**2
PLAT245_ALERT_2_C U(iso) H14       Smaller than U(eq) C14       by      0.016 Ang**2
PLAT245_ALERT_2_C U(iso) H177      Smaller than U(eq) N2        by      0.014 Ang**2
PLAT341_ALERT_3_C Low Bond Precision on  C-C Bonds ...............    0.00622 Ang.
PLAT352_ALERT_3_C Short  N-H (X0.87,N1.01A)  N2       - H177     .       0.75 Ang.
PLAT390_ALERT_3_C Deviating Methyl C14       X-C-H Bond Angle ....        116 Degree
PLAT480_ALERT_4_C Long H...A H-Bond Reported H3       ..N5               2.65 Ang.
PLAT480_ALERT_4_C Long H...A H-Bond Reported H6       ..N5               2.64 Ang.
PLAT480_ALERT_4_C Long H...A H-Bond Reported H7       ..O4               2.61 Ang.
PLAT480_ALERT_4_C Long H...A H-Bond Reported H10      ..O2               2.63 Ang.
PLAT480_ALERT_4_C Long H...A H-Bond Reported H30      ..N7               2.68 Ang.
PLAT480_ALERT_4_C Long H...A H-Bond Reported H26      ..N7               2.68 Ang.
PLAT905_ALERT_3_C Negative K value in the Analysis of Variance ...     -2.906 Report
PLAT911_ALERT_3_C Missing FCF Refl Between Thmin & STh/L=    0.595         20 Report
```

Alert level G

```
PLAT020_ALERT_3_G The Value of Rint is Greater Than 0.12 .........      0.141 Report
PLAT083_ALERT_2_G SHELXL Second Parameter in WGHT  Unusually Large      10.15 Why ?
PLAT164_ALERT_4_G Nr. of Refined C-H H-Atoms in Heavy-Atom Struct.         33 Note
PLAT793_ALERT_4_G Model has Chirality at N2          (Centro SPGR)          S Verify
PLAT793_ALERT_4_G Model has Chirality at N3          (Centro SPGR)          S Verify
PLAT793_ALERT_4_G Model has Chirality at N4          (Centro SPGR)          S Verify
PLAT794_ALERT_5_G Tentative Bond Valency for Cu1       (II)      .       2.08 Info
PLAT909_ALERT_3_G Percentage of I>2sig(I) Data at Theta(Max) Still        58% Note
PLAT910_ALERT_3_G Missing # of FCF Reflection(s) Below Theta(Min).          2 Note
PLAT933_ALERT_2_G Number of OMIT Records in Embedded .res File ...         21 Note
PLAT978_ALERT_2_G Number C-C Bonds with Positive Residual Density.          1 Info
```

```
   0 ALERT level A = Most likely a serious problem - resolve or explain
   0 ALERT level B = A potentially serious problem, consider carefully
  19 ALERT level C = Check. Ensure it is not caused by an omission or oversight
  11 ALERT level G = General information/check it is not something unexpected

   0 ALERT type 1 CIF construction/syntax error, inconsistent or missing data
   7 ALERT type 2 Indicator that the structure model may be wrong or deficient
  11 ALERT type 3 Indicator that the structure quality may be low
  11 ALERT type 4 Improvement, methodology, query or suggestion
   1 ALERT type 5 Informative message, check
```

It is advisable to attempt to resolve as many as possible of the alerts in all categories. Often the minor alerts point to easily fixed oversights, errors and omissions in your CIF or refinement strategy, so attention to these fine details can be worthwhile. In order to resolve some of the more serious problems it may be necessary to carry out additional measurements or structure refinements. However, the purpose of your study may justify the reported deviations and the more serious of these should normally be commented upon in the discussion or experimental section of a paper or in the "special_details" fields of the CIF. checkCIF was carefully designed to identify outliers and unusual parameters, but every test has its limitations and alerts that are not important in a particular case may appear. Conversely, the absence of alerts does not guarantee there are no aspects of the results needing attention. It is up to the individual to critically assess their own results and, if necessary, seek expert advice.

Publication of your CIF in IUCr journals

A basic structural check has been run on your CIF. These basic checks will be run on all CIFs submitted for publication in IUCr journals (*Acta Crystallographica*, *Journal of Applied Crystallography*, *Journal of Synchrotron Radiation*); however, if you intend to submit to *Acta Crystallographica Section C* or *E* or *IUCrData*, you should make sure that full publication checks are run on the final version of your CIF prior to submission.

Publication of your CIF in other journals

Please refer to the *Notes for Authors* of the relevant journal for any special instructions relating to CIF submission.

PLATON version of 30/01/2018; check.def file version of 30/01/2018

Datablock AT802_a - ellipsoid plot

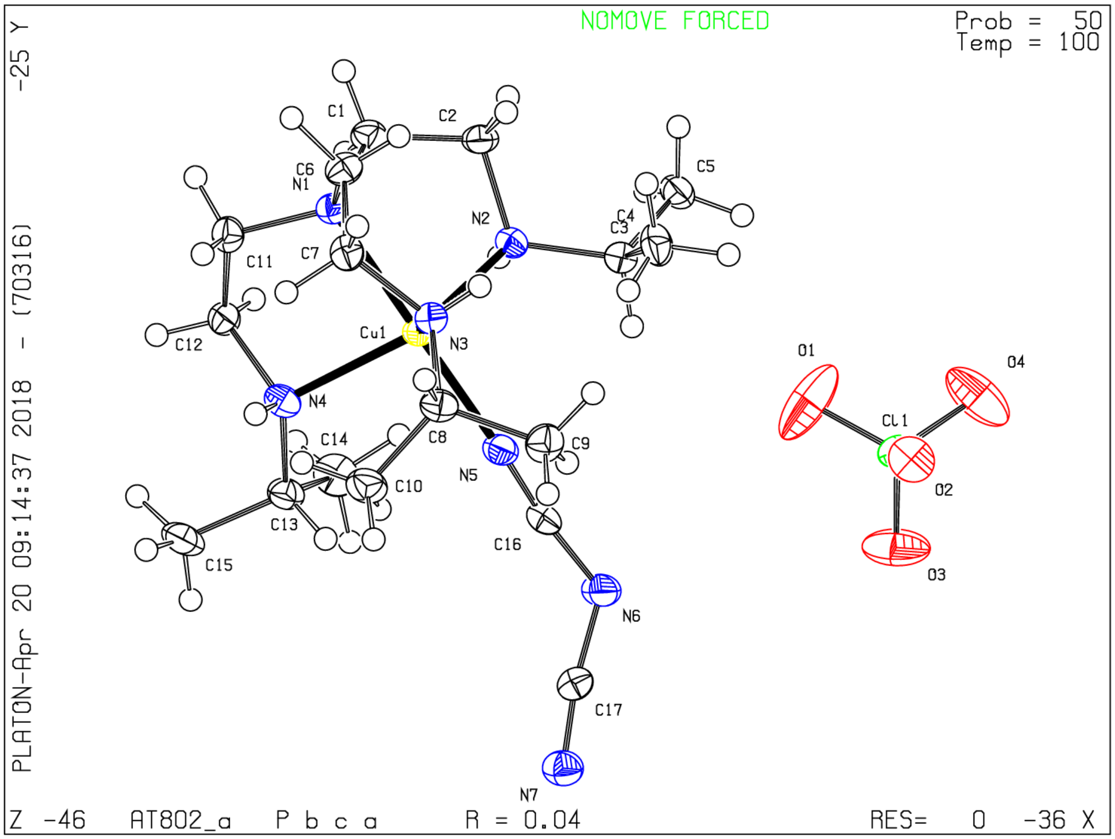

checkCIF/PLATON report

Structure factors have been supplied for datablock(s) rf956a

THIS REPORT IS FOR GUIDANCE ONLY. IF USED AS PART OF A REVIEW PROCEDURE FOR PUBLICATION, IT SHOULD NOT REPLACE THE EXPERTISE OF AN EXPERIENCED CRYSTALLOGRAPHIC REFEREE.

No syntax errors found.   CIF dictionary   Interpreting this report

Datablock: rf956a

```
Bond precision:  C-C = 0.0053 A         Wavelength=0.71073

Cell:           a=8.8023(7)    b=29.700(2)        c=11.2141(10)
                alpha=90       beta=93.260(4)     gamma=90
Temperature:    100 K

                Calculated                  Reported
Volume          2926.9(4)                   2926.9(4)
Space group     P 21/c                      P 21/c
Hall group      -P 2ybc                     -P 2ybc
Moiety formula  C23 H32 Cu N7 O2 S, Cl O4   C23 H32 Cu N7 O2 S, Cl O4
Sum formula     C23 H32 Cl Cu N7 O6 S       C23 H32 Cl Cu N7 O6 S
Mr              633.62                      633.60
Dx,g cm-3       1.438                       1.438
Z               4                           4
Mu (mm-1)       0.958                       0.958
F000            1316.0                      1316.0
F000'           1318.82
h,k,lmax        11,37,14                    11,37,14
Nref            6414                        6395
Tmin,Tmax       0.813,0.891                 0.776,0.935
Tmin'           0.802

Correction method= # Reported T Limits: Tmin=0.776 Tmax=0.935
AbsCorr = MULTI-SCAN

Data completeness= 0.997          Theta(max)= 27.025

R(reflections)= 0.0542( 4296)     wR2(reflections)= 0.1258( 6395)

S = 1.062                  Npar= 395
```

The following ALERTS were generated. Each ALERT has the format
test-name_ALERT_alert-type_alert-level.
Click on the hyperlinks for more details of the test.

Alert level C

```
PLAT242_ALERT_2_C Low    'MainMol' Ueq as Compared to Neighbors of         O1 Check
PLAT244_ALERT_4_C Low    'Solvent' Ueq as Compared to Neighbors of        Cl1 Check
PLAT410_ALERT_2_C Short Intra H...H Contact  H3A      ..H9A      .       1.99 Ang.
                                                    x,y,z  =      1_555 Check
PLAT601_ALERT_2_C Structure Contains Solvent Accessible VOIDS of .         37 Ang**3
PLAT906_ALERT_3_C Large K Value in the Analysis of Variance ......      2.743 Check
PLAT911_ALERT_3_C Missing FCF Refl Between Thmin & STh/L=    0.600          2 Report
```

Alert level G

```
PLAT003_ALERT_2_G Number of Uiso or Uij Restrained non-H Atoms ...          6 Report
PLAT186_ALERT_4_G The CIF-Embedded .res File Contains ISOR Records          2 Report
PLAT231_ALERT_4_G Hirshfeld Test (Solvent)   Cl1      --O3B      .        6.3 s.u.
PLAT302_ALERT_4_G Anion/Solvent/Minor-Residue Disorder (Resd  2  )        80% Note
PLAT432_ALERT_2_G Short Inter X...Y Contact  O3B      ..C2               3.01 Ang.
                                                    x,y,z  =      1_555 Check
PLAT794_ALERT_5_G Tentative Bond Valency for Cu1       (I)       .       1.19 Info
PLAT860_ALERT_3_G Number of Least-Squares Restraints .............         36 Note
PLAT883_ALERT_1_G No Info/Value for _atom_sites_solution_primary .     Please Do !
PLAT910_ALERT_3_G Missing # of FCF Reflection(s) Below Theta(Min).          3 Note
PLAT912_ALERT_4_G Missing # of FCF Reflections Above STh/L=  0.600         16 Note
PLAT933_ALERT_2_G Number of OMIT Records in Embedded .res File ...          4 Note
PLAT941_ALERT_3_G Average HKL Measurement Multiplicity ...........        3.9 Low
PLAT978_ALERT_2_G Number C-C Bonds with Positive Residual Density.          0 Info
```

---

0 **ALERT level A** = Most likely a serious problem - resolve or explain
0 **ALERT level B** = A potentially serious problem, consider carefully
6 **ALERT level C** = Check. Ensure it is not caused by an omission or oversight
13 **ALERT level G** = General information/check it is not something unexpected

1 ALERT type 1 CIF construction/syntax error, inconsistent or missing data
7 ALERT type 2 Indicator that the structure model may be wrong or deficient
5 ALERT type 3 Indicator that the structure quality may be low
5 ALERT type 4 Improvement, methodology, query or suggestion
1 ALERT type 5 Informative message, check

It is advisable to attempt to resolve as many as possible of the alerts in all categories. Often the minor alerts point to easily fixed oversights, errors and omissions in your CIF or refinement strategy, so attention to these fine details can be worthwhile. In order to resolve some of the more serious problems it may be necessary to carry out additional measurements or structure refinements. However, the purpose of your study may justify the reported deviations and the more serious of these should normally be commented upon in the discussion or experimental section of a paper or in the "special_details" fields of the CIF. checkCIF was carefully designed to identify outliers and unusual parameters, but every test has its limitations and alerts that are not important in a particular case may appear. Conversely, the absence of alerts does not guarantee there are no aspects of the results needing attention. It is up to the individual to critically assess their own results and, if necessary, seek expert advice.

Publication of your CIF in IUCr journals

A basic structural check has been run on your CIF. These basic checks will be run on all CIFs submitted for publication in IUCr journals (*Acta Crystallographica*, *Journal of Applied Crystallography*, *Journal of Synchrotron Radiation*); however, if you intend to submit to *Acta Crystallographica Section C* or *E* or *IUCrData*, you should make sure that full publication checks are run on the final version of your CIF prior to submission.

Publication of your CIF in other journals

Please refer to the *Notes for Authors* of the relevant journal for any special instructions relating to CIF submission.

PLATON version of 22/04/2020; check.def file version of 09/03/2020

Datablock rf956a - ellipsoid plot

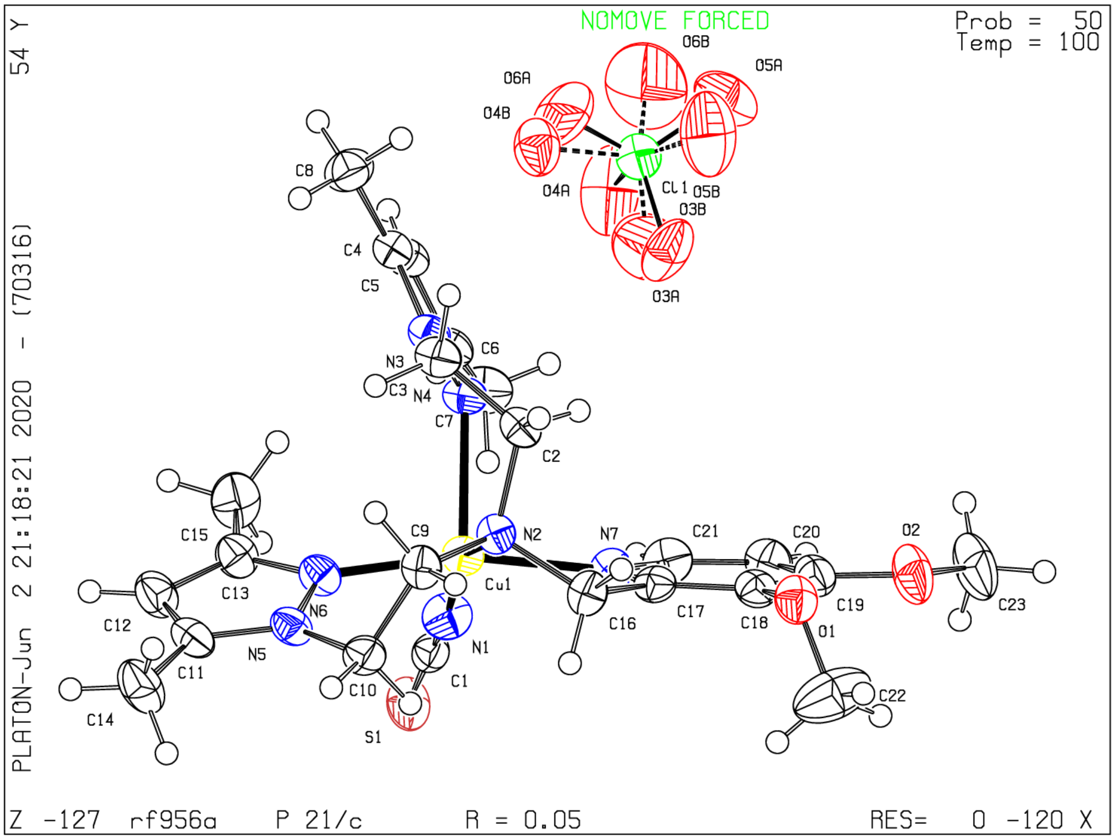

checkCIF/PLATON report

Structure factors have been supplied for datablock(s) rf1299a

THIS REPORT IS FOR GUIDANCE ONLY. IF USED AS PART OF A REVIEW PROCEDURE FOR PUBLICATION, IT SHOULD NOT REPLACE THE EXPERTISE OF AN EXPERIENCED CRYSTALLOGRAPHIC REFEREE.

No syntax errors found. CIF dictionary Interpreting this report

Datablock: rf1299a

```
Bond precision:  C-C = 0.0106 A          Wavelength=0.71073

Cell:           a=9.999(1)          b=15.7623(17)     c=15.9456(18)
                alpha=114.689(5)    beta=100.284(5)   gamma=103.717(5)
Temperature:    100 K
```

	Calculated	Reported
Volume	2105.8(4)	2105.7(4)
Space group	P 1	P 1
Hall group	P 1	P 1
Moiety formula	3(C23 H33 Cu N10), 3(Cl O4), 2(H2 O)	3(C23 H33 Cu N10), 3(Cl O4), 2(H2 O)
Sum formula	C69 H103 Cl3 Cu3 N30 O14	C69 H103 Cl3 Cu3 N30 O14
Mr	1873.81	1873.78
Dx,g cm-3	1.478	1.478
Z	1	1
Mu (mm-1)	0.925	0.925
F000	977.0	977.0
F000’	978.70	
h,k,lmax	13,21,21	13,20,21
Nref	20894[10447]	17329
Tmin,Tmax	0.847,0.903	0.794,0.932
Tmin’	0.839	

```
Correction method= # Reported T Limits: Tmin=0.794 Tmax=0.932
AbsCorr = MULTI-SCAN

Data completeness= 1.66/0.83       Theta(max)= 28.276

R(reflections)= 0.0549( 13745)     wR2(reflections)= 0.1347( 17329)

S = 1.044                  Npar= 1099
```

The following ALERTS were generated. Each ALERT has the format
test-name_ALERT_alert-type_alert-level.
Click on the hyperlinks for more details of the test.

Alert level C

```
PLAT220_ALERT_2_C NonSolvent Resd 1  N   Ueq(max) / Ueq(min) Range        3.2 Ratio
PLAT241_ALERT_2_C High   'MainMol' Ueq as Compared to Neighbors of         N9 Check
PLAT241_ALERT_2_C High   'MainMol' Ueq as Compared to Neighbors of        N29 Check
PLAT242_ALERT_2_C Low    'MainMol' Ueq as Compared to Neighbors of        C23 Check
PLAT341_ALERT_3_C Low Bond Precision on  C-C Bonds ...............    0.01064 Ang.
PLAT910_ALERT_3_C Missing # of FCF Reflection(s) Below Theta(Min).          5 Note
PLAT911_ALERT_3_C Missing FCF Refl Between Thmin & STh/L=    0.600         64 Report
PLAT915_ALERT_3_C No Flack x Check Done: Low Friedel Pair Coverage         71 %
PLAT975_ALERT_2_C Check Calcd Resid. Dens.  1.02A   From O14             0.41 eA-3
```

Alert level G

```
PLAT002_ALERT_2_G Number of Distance or Angle Restraints on AtSite          5 Note
PLAT003_ALERT_2_G Number of Uiso or Uij Restrained non-H Atoms ...          1 Report
PLAT007_ALERT_5_G Number of Unrefined Donor-H Atoms ..............          1 Report
PLAT154_ALERT_1_G The s.u.'s on the Cell Angles are Equal ..(Note)      0.005 Degree
PLAT172_ALERT_4_G The CIF-Embedded .res File Contains DFIX Records          1 Report
PLAT186_ALERT_4_G The CIF-Embedded .res File Contains ISOR Records          1 Report
PLAT244_ALERT_4_G Low    'Solvent' Ueq as Compared to Neighbors of        Cl1 Check
PLAT244_ALERT_4_G Low    'Solvent' Ueq as Compared to Neighbors of        Cl2 Check
PLAT343_ALERT_2_G Unusual sp?      Angle Range in Main Residue for        C23 Check
PLAT432_ALERT_2_G Short Inter X...Y Contact  O8       ..C31              2.98 Ang.
                                                    x,y,z  =      1_555 Check
PLAT790_ALERT_4_G Centre of Gravity not Within Unit Cell: Resd.  #          6 Note
              Cl O4
PLAT790_ALERT_4_G Centre of Gravity not Within Unit Cell: Resd.  #          8 Note
              H2 O
PLAT794_ALERT_5_G Tentative Bond Valency for Cu1       (I)       .       1.18 Info
PLAT794_ALERT_5_G Tentative Bond Valency for Cu2       (I)       .       1.19 Info
PLAT794_ALERT_5_G Tentative Bond Valency for Cu3       (I)       .       1.20 Info
PLAT860_ALERT_3_G Number of Least-Squares Restraints .............         12 Note
PLAT883_ALERT_1_G No Info/Value for _atom_sites_solution_primary .     Please Do !
PLAT912_ALERT_4_G Missing # of FCF Reflections Above STh/L=  0.600        418 Note
PLAT933_ALERT_2_G Number of OMIT Records in Embedded .res File ...          7 Note
PLAT965_ALERT_2_G The SHELXL WEIGHT Optimisation has not Converged     Please Check
PLAT978_ALERT_2_G Number C-C Bonds with Positive Residual Density.          0 Info
PLAT992_ALERT_5_G Repd & Actual _reflns_number_gt Values Differ by          2 Check
```

0 **ALERT level A** = Most likely a serious problem - resolve or explain
0 **ALERT level B** = A potentially serious problem, consider carefully
9 **ALERT level C** = Check. Ensure it is not caused by an omission or oversight
22 **ALERT level G** = General information/check it is not something unexpected

2 ALERT type 1 CIF construction/syntax error, inconsistent or missing data
12 ALERT type 2 Indicator that the structure model may be wrong or deficient
5 ALERT type 3 Indicator that the structure quality may be low
7 ALERT type 4 Improvement, methodology, query or suggestion
5 ALERT type 5 Informative message, check

It is advisable to attempt to resolve as many as possible of the alerts in all categories. Often the minor alerts point to easily fixed oversights, errors and omissions in your CIF or refinement strategy, so attention to these fine details can be worthwhile. In order to resolve some of the more serious problems it may be necessary to carry out additional measurements or structure refinements. However, the purpose of your study may justify the reported deviations and the more serious of these should normally be commented upon in the discussion or experimental section of a paper or in the "special_details" fields of the CIF. checkCIF was carefully designed to identify outliers and unusual parameters, but every test has its limitations and alerts that are not important in a particular case may appear. Conversely, the absence of alerts does not guarantee there are no aspects of the results needing attention. It is up to the individual to critically assess their own results and, if necessary, seek expert advice.

Publication of your CIF in IUCr journals

A basic structural check has been run on your CIF. These basic checks will be run on all CIFs submitted for publication in IUCr journals (*Acta Crystallographica*, *Journal of Applied Crystallography*, *Journal of Synchrotron Radiation*); however, if you intend to submit to *Acta Crystallographica Section C* or *E* or *IUCrData*, you should make sure that full publication checks are run on the final version of your CIF prior to submission.

Publication of your CIF in other journals

Please refer to the *Notes for Authors* of the relevant journal for any special instructions relating to CIF submission.

PLATON version of 04/06/2020; check.def file version of 02/06/2020

Datablock rf1299a - ellipsoid plot

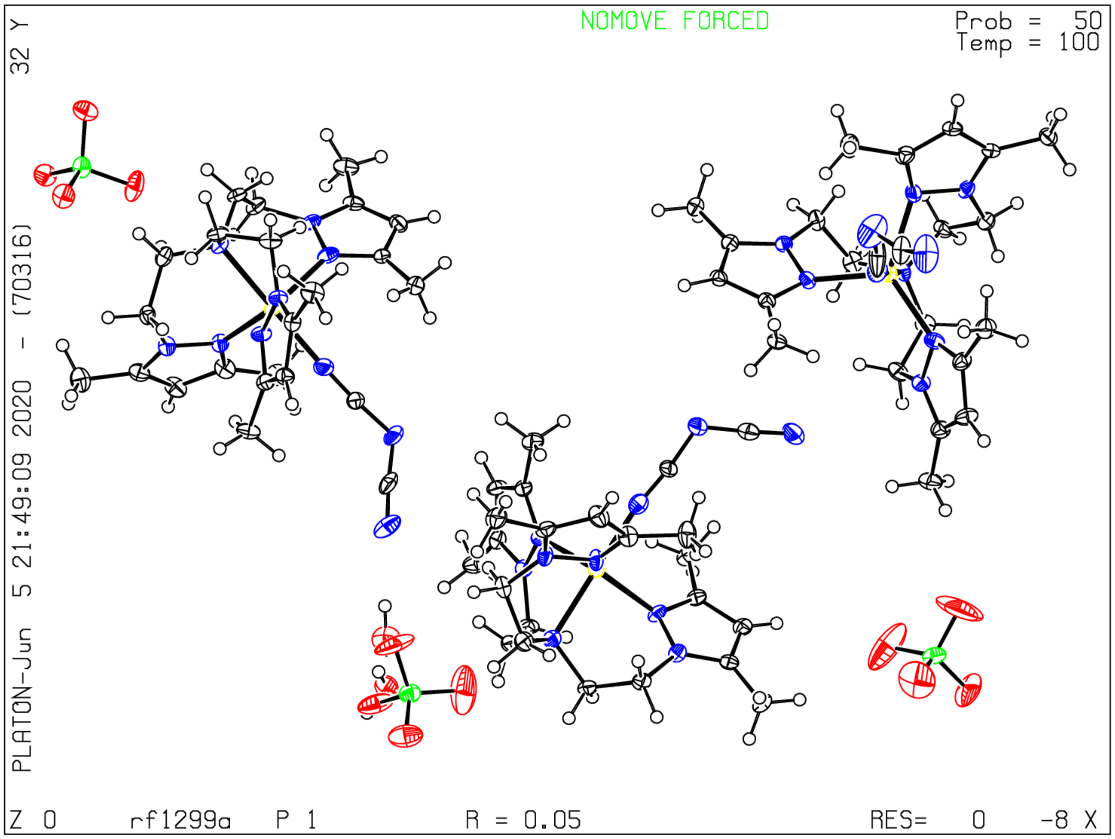

Supplement: Supplementary file 1 [file molecules-25-03376-s001.zip › molecules-875219-final-SM/Binder-HKL_checkcif files.pdf]
