# Supplementary material for: Five-Coordinated Geometries from Molecular Structures to Solutions in Copper(II) Complexes Generated from Polydentate-N-Donor Ligands and Pseudohalides
Source: Molecules. 2020 Jul 25;25(15):3376. doi: 10.3390/molecules25153376 (PMC7436159; doi:10.3390/molecules25153376)
Supplement: Supplementary file 1 [file molecules-25-03376-s001.zip › molecules-875219-final-SM/Supplementary Material.pdf]

SUBLEMENTARY MATERIALS

Five-coordinated Geometries from Molecular Structures to Solutions in Copper(II) Complexes Generated from Polydentate-*N*-donor Ligands and Pseudohalides

Franz A. Mautner [1,*], Roland C. Fischer [2], Ana Torvisco [2], Maher M. Henary [3], Febee R. Louka [4], Salah S. Massoud [4,5*], Nahed M. H. Salem [5]

[1] *Institut für Physikalische und Theoretische Chemie, Technische Universität Graz, Stremayrgasse 9, A-8010 Graz, Austria*

[2] *Institut für Anorganische Chemie, Technische Universität Graz, Stremayrgasse 9, A-8010 Graz, Austria*

[3] *Department of Chemistry and Biochemistry, University of California, Los Angeles, CA 90095-1569, U.S.A.* henary@chem.ucla.edu

[4] *Department of Chemistry, University of Louisiana at Lafayette, P.O. Box 43700 Lafayette, LA 70504, U.S.A.*

[5] *Department of Chemistry, Faculty of Science, Alexandria University, Moharam Bey, Alexandria, Egypt*

Table of Contents page

Figures S1-S6. packing plots for compounds **1** – **6.** 2-4
Figures S7 and **S8**. Coordination figures of Cu1 and Cu3 of **6.** 5
Figure S9. UV-Vis spectrum of complex $[Cu(L^1)(NCS)_2]$ (**1**) in acetonitrile. 6
Figure S10. UV-Vis spectrum of complex $[Cu(L^2)(NCS)_2]$ (**2**) in acetonitrile. 7
Figure S11. UV-Vis spectrum of complex $[Cu(isp_3tren)(N_3)]ClO_4$ (**3**) in acetonitrile. 8
Figure S12. UV-Vis spectrum of complex $[Cu(isp_3tren)(dca)]ClO_4$ (**4**) in acetonitrile. 9
Figure S13. UV-Vis spectrum of complex $[Cu(L^3)(NCS)]ClO_4$ (**5**) in acetonitrile. 10
Figure S14. UV-Vis spectrum of complex $[Cu(tedmpza)(dca)]ClO_4·0.67H_2O$ (**6**) in acetonitrile. 11
Figure S15. UV-Vis spectrum of complex $[Cu(L^4)(dca)](ClO_4)·2H_2O$ (**7**) in acetonitrile. 12
Tables S1-S3. Bond parameters of **1**, **2**, **5**, and **3**, **4**, respectively and **6**. 13-15
Table S4. Possible hydrogen bonds for compounds **1** – **6** 16, 17

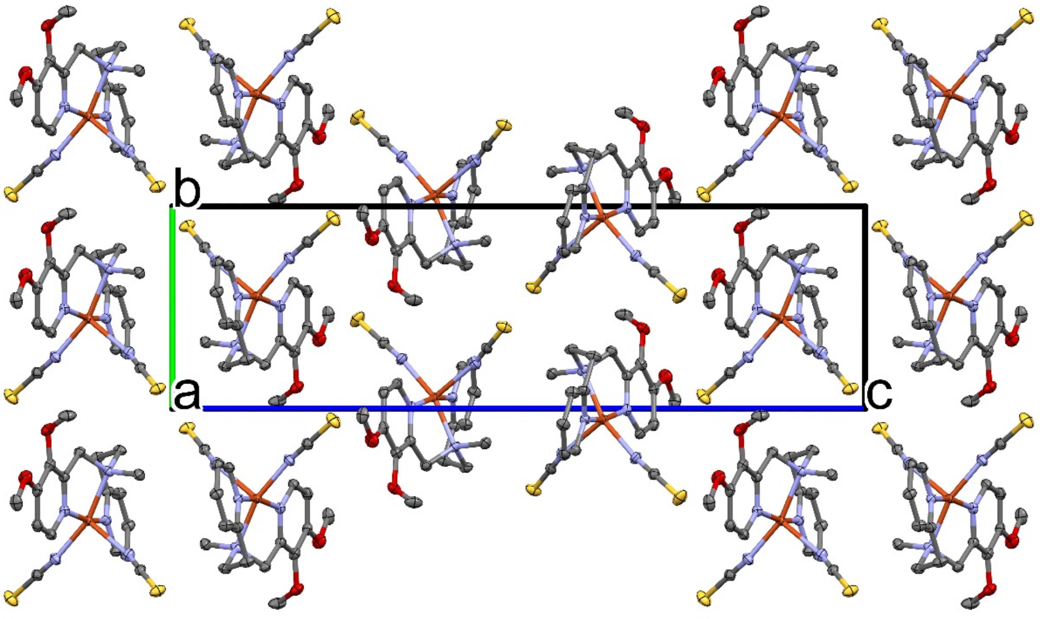

Figure S1. Packing plot of **1**.

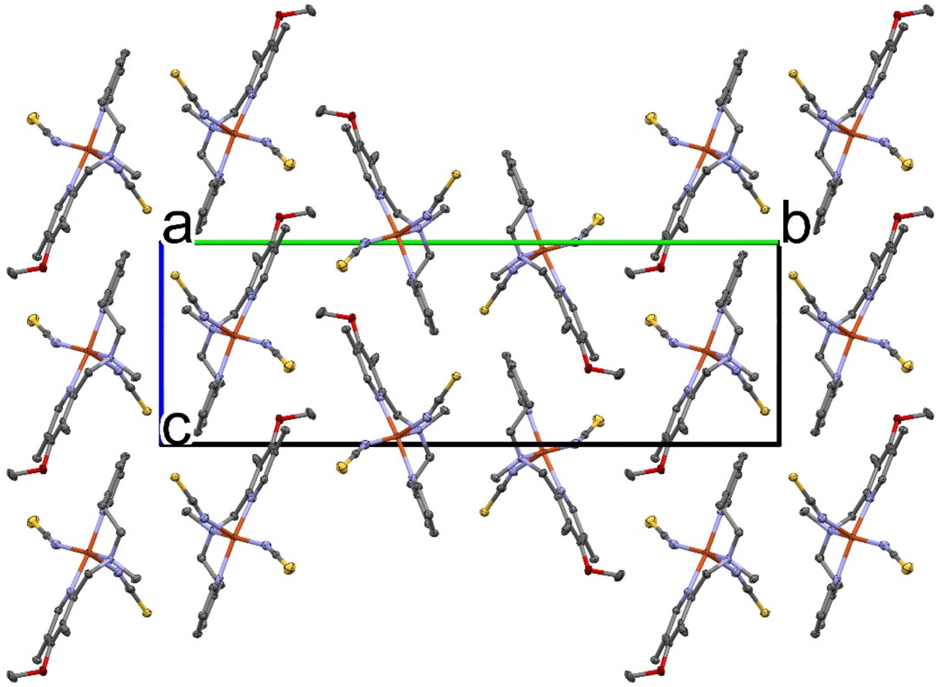

Figure S2. Packing plot of **2**.

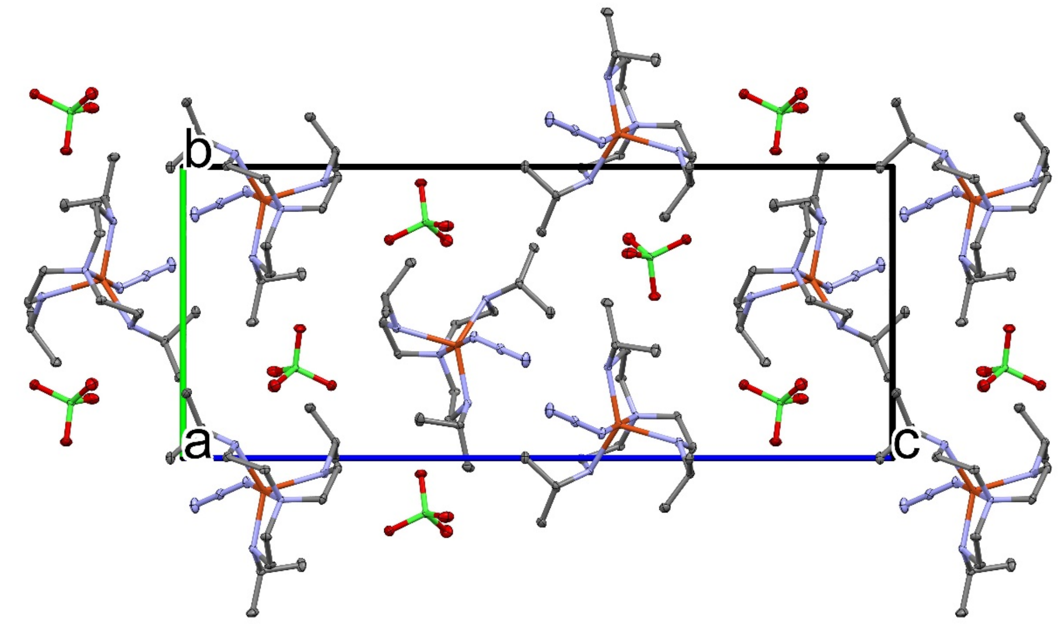

Figure S3. Packing plot of **3**.

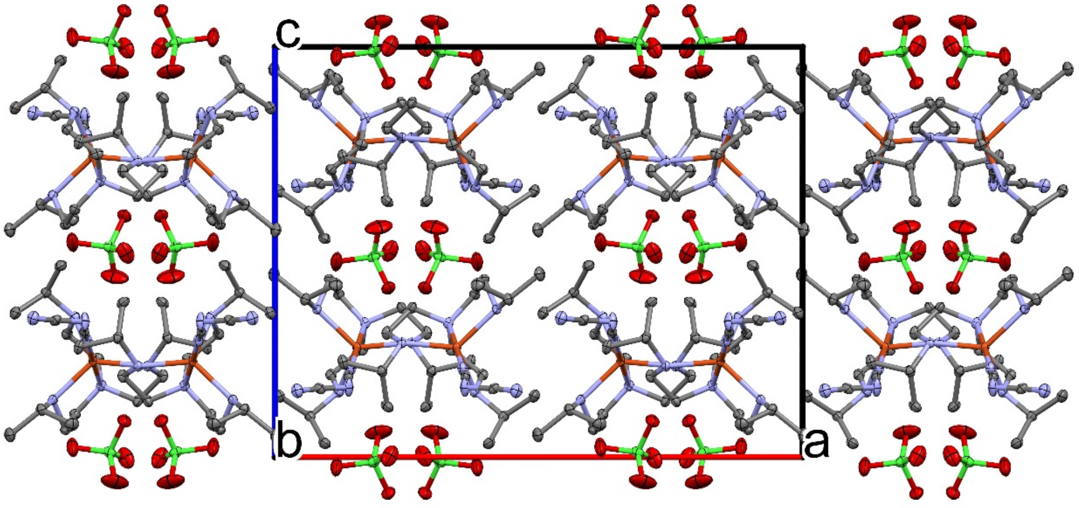

Figure S4. Packing plot of **4**.

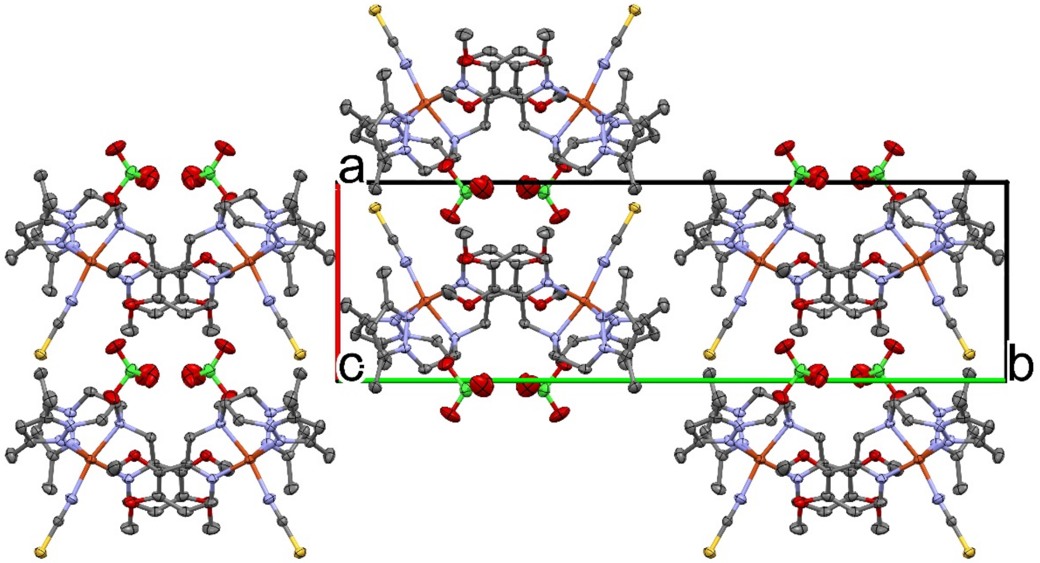

Figure S5. Packing plot of **5**.

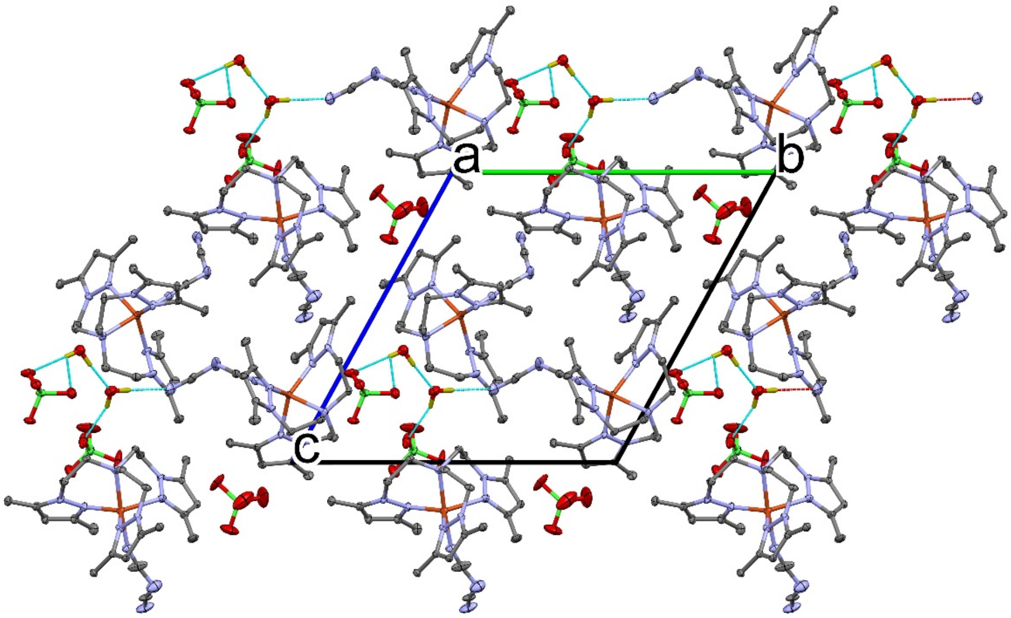

Figure S6. Packing plot of **6**. (Broken lines indicate hydrogen bonds)

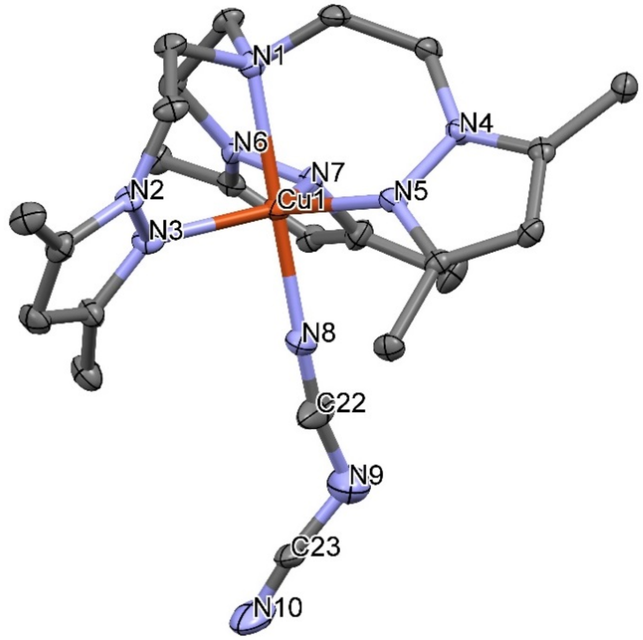

Figure S7. Coordination figure of Cu1 in **6**.

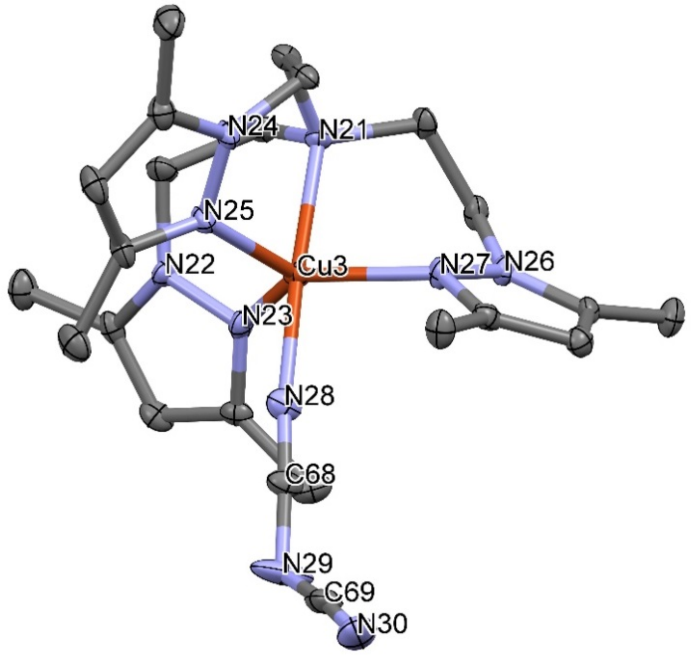

Figure S8. Coordination figure of Cu3 in **6**.

Spectrum/Peak Report Date 10/21/2015 Time 13:14:11 Page 1 of 1

Method file : TEST.M (modified) Last update: Date 10/21/2015 Time 1:13:44 PM
Information : Default Method
Data File : <untitled>

Overlaid Spectra:

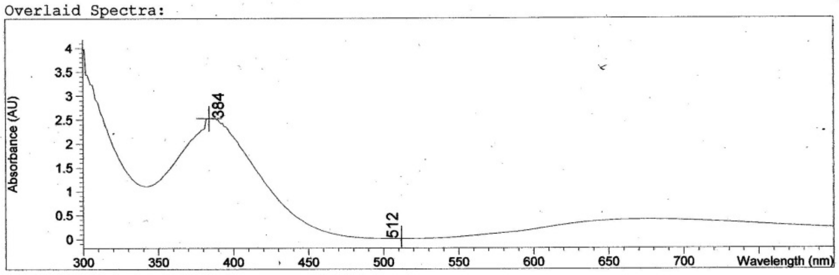

#	Name	Peaks(nm)	Abs(AU)	Valleys(nm)	Abs(AU)
1		384.0	2.52570	512.0	1.6904E-2

Report generated by : Diana Signature: ....

*** End Spectrum/Peak Report ***

#2955

0.0100 g /10 mL CH3CN

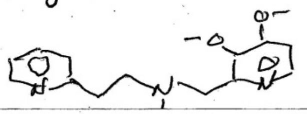

/Cu(ClO4)2 / NH4NCS

Spectrum/Peak Report Date 10/21/2015 Time 13:16:10 Page 1 of 1

Method file : TEST.M (modified) Last update: Date 10/21/2015 Time 1:16:01 PM
Information : Default Method
Data File : <untitled>

Overlaid Spectra:

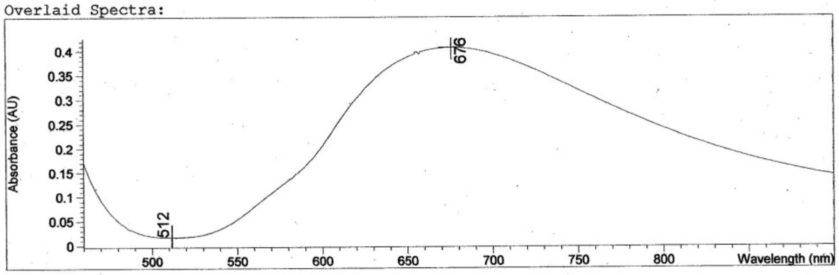

#	Name	Peaks(nm)	Abs(AU)	Valleys(nm)	Abs(AU)
1		676.0	0.40730	512.0	1.6904E-2

Report generated by : Diana Signature: ....

*** End Spectrum/Peak Report ***

Figure S9. UV-Vis spectrum of complex [Cu(L^1)$(NCS)_2$] (**1**) in acetonitrile solution.

Spectrum/Peak Report Date 10/21/2015 Time 13:26:49 Page 1 of 1

Method file : TEST.M (modified) Last update: Date 10/21/2015 Time 1:26:37 PM

Information : Default Method

Data File : <untitled>

Overlaid Spectra:

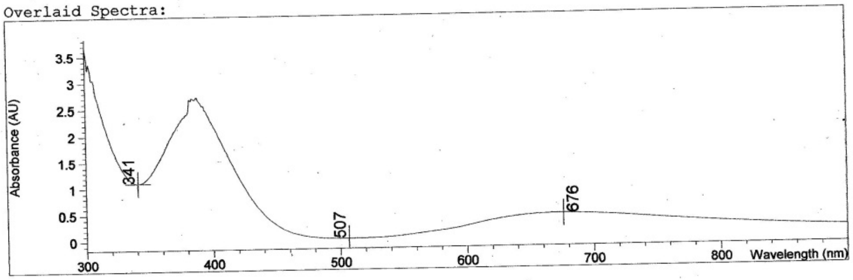

#	Name	Peaks (nm)	Abs (AU)	Valleys (nm)	Abs (AU)
1		676.0	0.43721	507.0	2.0803E-2
1		***	***	341.0	1.09770
		388	2.714		

Report generated by : Diana Signature: ....

*** End Spectrum/Peak Report ***

#2949

0.0164g /10 mL CH3CN

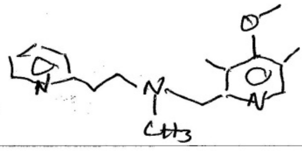

| Cu(ClO4)2 | NH4NCS

Spectrum/Peak Report Date 10/21/2015 Time 13:28:41 Page 1 of 1

Method file : TEST.M (modified) Last update: Date 10/21/2015 Time 1:28:35 PM

Information : Default Method

Data File : <untitled>

Overlaid Spectra:

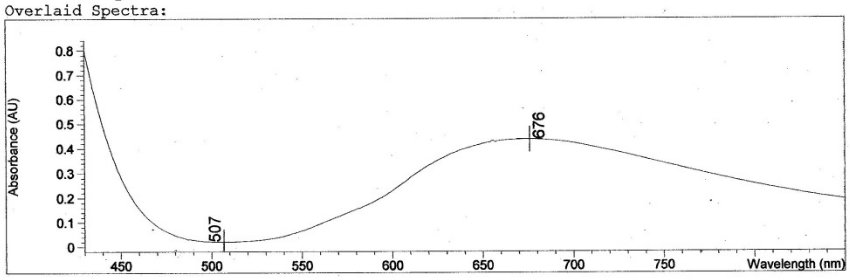

#	Name	Peaks (nm)	Abs (AU)	Valleys (nm)	Abs (AU)
1		676.0	0.43721	507.0	2.0803E-2
1		***	***	***	***

Report generated by : Diana Signature: ....

*** End Spectrum/Peak Report ***

Figure S10. UV-Vis spectra of complex [Cu(L^2)$(NCS)_2$] (**2**) in acetonitrile solution.

Spectrum/Peak Report Date 3/28/2018 Time 13:10:41 Page 1 of 1

#3234

Method file : TEST.M (modified) Last update: Date 3/28/2018 Time 1:10:25 PM
Information : Default Method
Data File : C:\HPCHEM\1\DATA\3234.SD Created : 3/28/18 13:10:11

Overlaid Spectra:

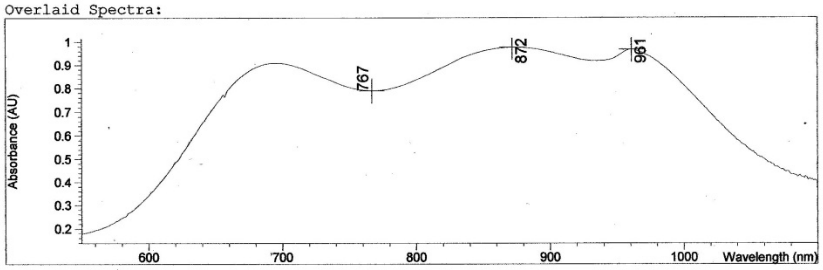

#	Name	Peaks(nm)	Abs(AU)	Valleys(nm)	Abs(AU)
1		872.0	0.97569	767.0	0.78910
1		961.0	0.96573	***	***

Report generated by : Ada Signature: ....

*** End Spectrum/Peak Report ***

0.0104g in 10mL AN

Figure S11. UV-Vis spectra of complex [Cu(isp$_3$tren)(N_3)]ClO_4 (**3**) in acetonitrile solution.

Spectrum/Peak Report Date 3/26/2018 Time 10:03:10 Page 1 of 1

#3245

Method file : TEST.M (modified) Last update: Date 3/26/2018 Time 10:01:58 AM
Information : Default Method
Data File : C:\HPCHEM\1\DATA\3245AN.SD Created : 3/26/18 10:04:43

Overlaid Spectra:

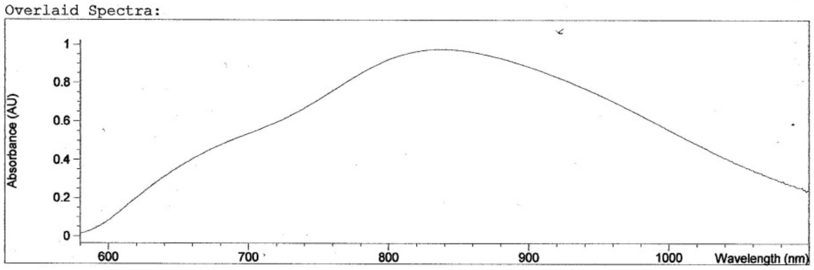

#	Name	Peaks(nm)	Abs(AU)	Valleys(nm)	Abs(AU)
1		***	***	***	***

Report generated by : Eamon Signature: ....

*** End Spectrum/Peak Report ***

#3245 0.0122g/10mL AN

λ	Abs
673	0.454598
837	0.975066

no peaks before 600nm

Figure S12. UV-Vis spectra of complex [Cu(isp$_3$tren)(dca)]ClO_4 (**4**) in acetonitrile solution.

Spectrum/Peak Report Date 4/21/2016 Time 15:46:26 Page 1 of 1

Method file : TEST.M (modified) Last update: Date 4/21/2016 Time 3:46:23 PM
Information : Default Method
Data File : <untitled>

Overlaid Spectra:

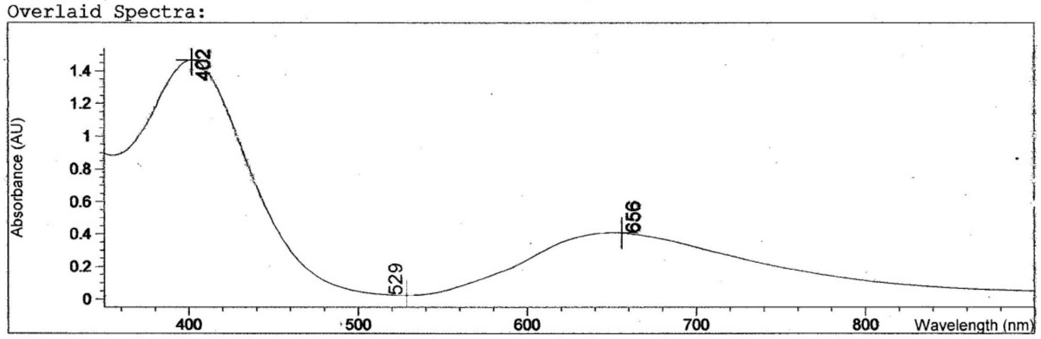

#	Name	Peaks(nm)	Abs(AU)	Valleys(nm)	Abs(AU)
1		402.0	1.46690	529.0	2.5256E-2
1		656.0	0.40735	***	***

Report generated by : Diana Signature: ....

*** End Spectrum/Peak Report ***

303 9
0.004 g/5 mL CH3CN

Cu(ClO4)2/

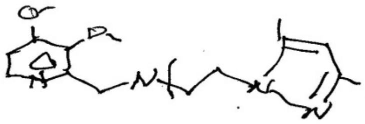

/NH4NCS

Figure S13. UV-Vis spectrum of complex [Cu(L^3)(NCS)]ClO_4 (**5**) in acetonitrile solution.

```
==========================================================================
Fixed Wavelength Report                 Date 3/12/2012 Time 12:07:05  Page 1 of 1
==========================================================================
```

#2322   0.0110g/10mL CH_3CN $Cu(ClO_4)_2$ / N-[illegible] Nadca

```
Method file :        <untitled>
Information :        Default Method
Data File   :        C:\HPCHEM\1\DATA\2322AN.SD      Created : 3/12/12 12:11:06

Overlaid Spectra:
```

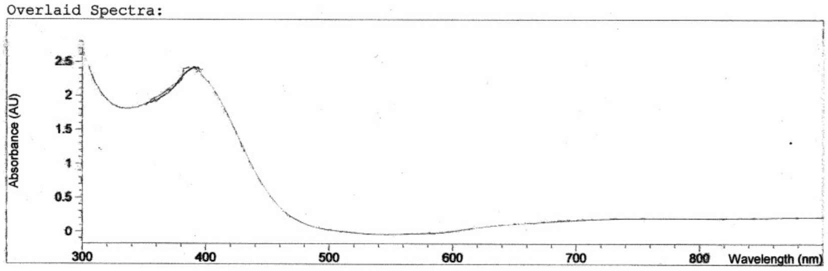

#	Name	Abs<480nm>
1		0.11928

Report generated by : Emily   Signature: ....

*** End Fixed Wavelength Report ***

No peaks before 300

378   2.185396

```
==========================================================================
Fixed Wavelength Report                 Date 3/12/2012 Time 12:09:29  Page 1 of 1
==========================================================================
```

#2322

```
Method file :        <untitled>
Information :        Default Method
Data File   :        C:\HPCHEM\1\DATA\2322AN.SD      Created : 3/12/12 12:11:06

Overlaid Spectra:
```

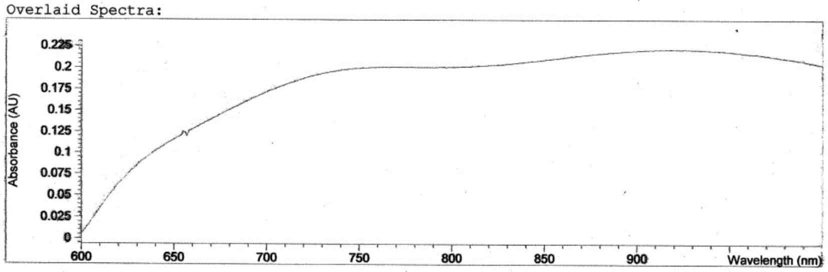

#	Name	Abs<480nm>
1		0.11928

Report generated by : Emily   Signature: ....

*** End Fixed Wavelength Report ***

921   0.221001

734   0.194994

Figure S14. UV-Vis spectrum of complex [Cu(tedmpza)(dca)]$ClO_4\cdot0.67H_2O$ (**6**) in acetonitrile solution.

```
================================================================================
Fixed Wavelength Report                    Date 4/18/2012 Time 13:06:46  Page 1 of 1
================================================================================
```

MD76 0.0048g /5mL CH_3CN

```
Method file :        <untitled>
Information :        Default Method
Data File   :        C:\HPCHEM\1\DATA\MD76AN.SD     Created : 4/18/12 13:05:32

Overlaid Spectra:
```

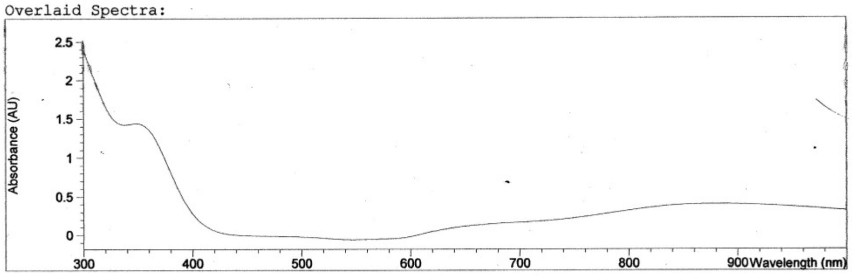

#	Name	Abs<480nm>
1		-1.7168E-2

Report generated by : Emily   Signature: ....

*** End Fixed Wavelength Report ***

```
================================================================================
Fixed Wavelength Report                    Date 4/18/2012 Time 13:07:07  Page 1 of 1
================================================================================

Method file :        <untitled>
Information :        Default Method
Data File   :        C:\HPCHEM\1\DATA\MD76AN.SD     Created : 4/18/12 13:05:32

Overlaid Spectra:
```

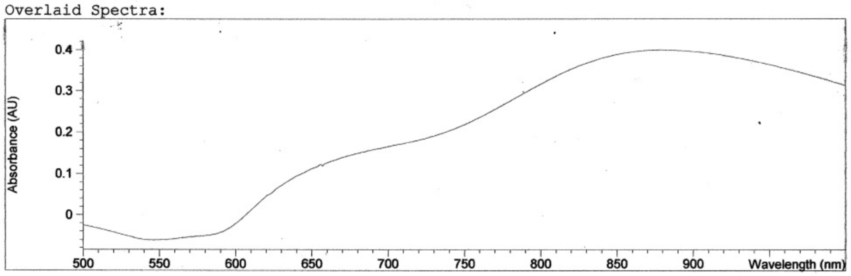

#	Name	Abs<480nm>
1		-1.7168E-2

Report generated by : Emily   Signature: ....

*** End Fixed Wavelength Report ***

665	0.132492
879	0.399626

Figure S15. UV-Vis spectrum of complex $[Cu(L^4)(dca)](ClO_4)\cdot 2H_2O$ (**7**) in acetonitrile solution.

Table S1. Selected bond distances (Å) and bond angles (°) of **1**, **2** and **5**.

Compound **1**			
Cu1-N1	1.972(2)	Cu1-N4	2.068(2)
Cu1-N3	2.010(2)	Cu1-N2	2.188(2)
Cu1-N5	2.018(2)	S2-C2	1.630(3)
N1-C2	1.157(3)	S1-C1	1.635(3)
N2-C1	1.157(3)		
N1-Cu1-N4	162.13(9)	N3-Cu1-N5	167.20(8)
Cu1-N1-C2	155.7(2)	Cu1-N2-C1	149.0(2)
N1-C2-S2	178.3(2)	N2-C1-S1	178.4(2)
Compound **2**			
Cu2-N2	1.988(6)	Cu2-N4	2.080(6)
Cu2-N3	2.005(5)	Cu2-N1	2.165(7)
Cu2-N5	2.006(5)	S2-C2	1.649(8)
N1-C1	1.153(9)	S1-C1	1.635(8)
N2-C2	1.163(10)		
N2-Cu2-N4	160.3(3)	N3-Cu2-N5	168.4(3)
Cu2-N1-C1	160.7(6)	Cu2-N2-C2	153.6(6)
N1-C1-S1	178.8(7)	N2-C2-S2	177.9(7)
Compound **5**			
Cu1-N1	1.955(3)	Cu1-N2	2.087(3)
Cu1-N6	2.007(3)	Cu1-N4	2.232(3)
Cu1-N7	2.024(3)	S1-C1	1.626(4)
N1-C1	1.155(4)		
N1-Cu1-N2	162.46(12)	N6-Cu1-N7	163.94(12)
Cu1-N1-C1	175.0(3)	N1-C1-S1	179.5(4)

Table S2. Selected bond distances (Å) and bond angles (°) of **3** and **4**.

Compound **3**			
Cu1-N5	1.9735(17)	Cu1-N2	2.1108(17)
Cu1-N1	2.0617(17)	Cu1-N4	2.2417(17)
Cu1-N3	2.0925(16)	N5-N6	1.193(3)
N6-N7	1.164(3)		
N5-Cu1-N1	179.28(7)	N2-Cu1-N3	132.54(6)
Cu1-N5-N6	130.37(14)	N5-N6-N7	176.3(2)
Compound **4**			
Cu1-N5	1.964(3)	Cu1-N4	2.125(3)
Cu1-N1	2.041(3)	Cu1-N3	2.199(3)
Cu1-N2	2.092(3)	N5-C16	1.149(5)
N6-C16	1.294(5)	N7-C17	1.155(5)
N6-C17	1.320(5)		
N1-Cu1-N5	176.21(14)	N2-Cu1-N4	129.85(14)
Cu1-N5-C16	171.9(3)	N5-C16-N6	173.3(4)
C16-N6-C17	122.1(4)	N6-C17-N7	171.8(4)

Table S3. Selected bond distances (Å) and bond angles (°) of **6**.

Compound **6**			
Cu1-N8	1.961(6)	Cu1-N1	2.094(5)
Cu1-N3	2.054(5)	Cu1-N7	2.110(5)
Cu1-N5	2.065(5)	N8-C22	1.145(10)
N9-C22	1.339(11)	N10-C23	1.107(10)
N9-C23	1.326(11)		
N1-Cu1-N8	176.0(2)	N3-Cu1-N7	128.2(2)
Cu1-N8-C22	162.5(7)	N8-C22-N9	167.8(11)
C22-N9-C23	116.1(8)	N9-C23-N10	163.5(9)
Cu2-N18	1.951(6)	Cu2-N11	2.082(5)
Cu2-N17	2.062(5)	Cu2-N13	2.109(5)
Cu2-N15	2.071(6)	N18-C45	1.161(9)
N19-C45	1.290(9)	N20-C46	1.147(9)
N19-C46	1.319(10)		
N11-Cu2-N18	178.4(2)	N15-Cu2-N17	125.3(2)
Cu2-N18-C45	172.3(6)	N18-C45-N19	174.2(7)
C45-N19-C46	118.2(6)	N19-C46-N20	175.9(8)
Cu3-N28	1.971(5)	Cu3-N25	2.079(5)
Cu3-N23	2.043(6)	Cu3-N21	2.097(5)
Cu3-N27	2.064(5)	N28-C68	1.162(8)
N29-C68	1.295(9)	N30-C69	1.153(9)
N29-C697	1.306(10)		
N21-Cu3-N28	176.7(2)	N23-Cu3-N27	127.8(2)
Cu3-N28-C68	173.6(6)	N28-C68-N29	175.5(7)
C68-N29-C69	121.0(6)	N29-C69-N30	174.7(8)

Table S4. Possible hydrogen bonds for compounds **1 - 6**
[Bond distances (Å) and bond angles (°), D = donor, A = acceptor]

Compound 1

D-H	d(D-H)	d(H..A)	<DHA	d(D..A)	A
C16-H16	0.950	2.917	137.47	3.673	S1 [-x+1, -y+3, -z+1]
C9-H9B	0.980	2.952	145.01	3.796	S2 [-x, y-1/2, -z+1/2]
C9-H9C	0.980	2.469	150.50	3.356	O2 [-x, y+1/2, -z+1/2]
C8-H8C	0.980	2.366	117.55	2.950	O1
C18-H18A	0.980	2.816	159.47	3.749	S1 [x, y-1, z]

Compound 2

D-H	d(D-H)	d(H..A)	<DHA	d(D..A)	A
C4-H4	0.950	2.997	128.43	3.664	S1 [x, y, z+1]
C10-H10B	0.990	2.913	170.07	3.892	S2 [x+1, y, z]
C19-H19C	0.980	2.902	150.02	3.782	S1 [x+1, y, z]

Compound 3

Domor	-H……	A	code	D-H	H…A	D….A……	D-H…A
N2	--H2	..O3	[4655]	1.00	2.26	3.162(2)	149
N3	--H3	..N7	[3556]	1.00	2.15	3.114(3)	162
N4	--H4	..O1	[1555]	1.00	2.21	3.101(2)	147
C1	--H1B	..N6	[1655]	0.99	2.62	3.530(2)	153
C5	--H5A	..N6	[ ]	0.98	2.52	3.245(3)	130
C8	--H8	..N6	[ ]	1.00	2.58	3.314(3)	130
C12	--H12A	.O1	[4655]	0.99	2.60	3.515(3)	154
C13	--H13	.O4	[1555]	1.00	2.51	3.237(3)	129
C14	--H14C	..N5	[ ]	0.98	2.49	3.336(3)	145

[4655.] = [3_655] = 1-x,1/2+y,1/2-z
[3556.] = [4_556] = 1/2+x,1/2-y,1-z
[1655.] = [1_655] = 1+x,y,z

Compound 4

Domor	-H……	A	code	D-H	H…A	D….A……	D-H…A
N4	--H2	..N7	[4655]	0.79(6)	2.56(5)	3.295(5)	157(4)
N3	--H24	..O3	[7655]	0.80(4)	2.55(4)	3.294(5)	155(4)
N2	--H177	.O2	[8554]	0.75(4)	2.38(4)	3.113(4)	166(4)
C2	--H5	. .N6	[7655]	1.00(5)	2.58(5)	3.471(6)	148(4)
C8	--H7	.. O4	[7655]	0.99(5)	2.60(4)	3.349(6)	132(3)
C1	--H11	..O2	[2664]	0.96(4)	2.59(4)	3.429(5)	146(3)
C11	--H22	..N7	[4655.]	0.98(4)	2.59(4)	3.399(5)	140(3)
C1	--H23	..O3	[8554]	0.90(5)	2.52(5)	3.334(6)	150(4)
C4	--H32	..O1	[1555]	0.98(5)	2.50(5)	3.125(6)	121(3)
C14	--H34	..O2	[8554]	1.01(6)	2.55(6)	3.397(7)	141(5)

[8554.] = [7_565] = x,1/2-y,-1/2+z
[4655.] = [3_655] = 1-x,1/2+y,1/2-z
[2664.] = [2_664] = 3/2-x,1-y,-1/2+z
[7655.] = [8_765] = 3/2-x,1/2+y,z

Compound 5

D-H	d(D-H)	d(H..A)	<DHA	d(D..A)	A
C2-H2A	0.990	2.437	116.02	3.006	O3B
C3-H3A	0.990	2.761	161.30	3.712	S1 [x+1, y, z]
C3-H3B	0.990	2.460	157.04	3.394	O4B
C7-H7A	0.980	2.447	151.33	3.340	N1

C9-H9B	0.990	2.497	166.64	3.468	O3A [x, -y+3/2, z-1/2]
C8-H8C	0.980	2.618	150.21	3.503	O4B
C10-H10A	0.990	2.468	122.99	3.119	O1 [x, -y+3/2, z-1/2]
C10-H10B	0.990	2.463	172.93	3.447	O6B [x, y, z-1]
C12-H12	0.950	3.022	140.34	3.802	S1 [-x+1, -y+1, -z]
C14-H14A	0.980	2.947	155.82	3.861	S1 [-x+1, -y+1, -z]
C16-H16A	0.990	2.527	163.83	3.489	O5A [x, -y+3/2, z-1/2]
C16-H16A	0.990	2.604	133.02	3.359	O3B [x, -y+3/2, z-1/2]
C20-H20	0.950	2.534	172.77	3.479	O3A [x-1, y, z]
C20-H20	0.950	2.583	128.11	3.257	O4B [x-1, y, z]
C22-H22B	0.980	2.593	138.55	3.391	O6A [x-1, -y+3/2, z-1/2]
C23-H23A	0.980	2.366	151.06	3.258	O5A [x-1, -y+3/2, z-1/2]
C23-H23A	0.980	2.617	133.75	3.370	O6B [x-1, -y+3/2, z-1/2]
C23-H23C	0.980	2.571	138.96	3.372	O5B [x-1, y, z]

Compound 6

D-H	d(D-H)	d(H..A)	<DHA	d(D..A)	A
C1-H1A	0.990	2.455	151.03	3.356	O12 [x+1, y-1, z-1]
C2-H2A	0.990	2.478	123.25	3.132	N20
C2-H2B	0.990	2.626	135.03	3.399	O10 [x+1, y-1, z-1]
C6-H6C	0.980	2.628	124.75	3.287	O4
C9-H9A	0.990	2.639	131.06	3.373	N7
C11-H11	0.950	2.589	150.04	3.445	N10 [x-1, y, z]
O13-H91	0.831	2.014	175.15	2.843	N30
O13-H92	0.837	2.968	166.12	3.786	Cl2
O13-H92	0.837	2.072	159.13	2.869	O6
O14-H93	0.837	1.955	161.12	2.761	O13
O14-H94	0.827	2.398	179.65	3.224	O11 [x, y-1, z]
C16-H16A	0.990	2.531	141.58	3.364	O6 [x+1, y, z-1]
C16-H16B	0.990	2.657	167.58	3.631	N30 [x+1, y, z-1]
C20-H20A	0.980	2.446	159.01	3.379	O5 [x+1, y, z-1]
C24-H24A	0.990	2.408	148.41	3.293	O9 [x+1, y-1, z]
C29-H29B	0.980	2.453	163.48	3.404	O14 [x+1, y, z]
C31-H31A	0.990	2.523	165.62	3.491	O12 [x+1, y-1, z]
C43-H43A	0.980	2.676	155.23	3.590	N20 [x-1, y, z]
C48-H48A	0.990	2.536	119.01	3.138	N10 [x-2, y, z]
C48-H48B	0.990	2.509	147.95	3.389	O14 [x, y+1, z]
C50-H50	0.950	2.677	139.22	3.452	N9 [x-1, y, z]
C52-H52C	0.980	2.693	140.93	3.509	N10 [x-2, y, z]
C54-H54A	0.990	2.475	152.58	3.385	O11
C55-H55A	0.990	2.673	133.05	3.426	N27
C61-H61A	0.990	2.449	164.70	3.414	O6 [x, y+1, z]
C62-H62A	0.990	2.540	150.59	3.437	O9 [x+1, y, z]
C62-H62B	0.990	2.603	138.63	3.410	O7 [x, y+1, z]
C66-H66A	0.980	2.621	166.88	3.583	O10 [x+1, y, z]
C66-H66C	0.980	2.690	142.66	3.520	N20 [x, y+1, z+1]
